# Interference of endogenous benzoic acid with the signatures of sulfonic acid derivatives and carbohydrates in fermented dairy products

**DOI:** 10.1016/j.fmre.2022.09.033

**Published:** 2022-11-09

**Authors:** Wei Jia, Xin Wang, Lin Shi

**Affiliations:** a School of Food and Biological Engineering, Shaanxi University of Science & Technology, Xi'an 710021, China; b Shaanxi Research Institute of Agricultural Products Processing Technology, Xi'an 710021, China

**Keywords:** Benzoic acid, Health effects, Fermented goat milk, Nutritional quality, Metabolomics and proteomics, UHPLC-Q-Orbitrap HRMS

## Abstract

Endogenous benzoic acid causes detrimental effects on public health, but the underlying mechanisms often remain elusive. Benzoic acid (0.00–40.00 mg *L*^−1^) was detected from sixty fermented goat milk samples in six replicates, indicating the existence of endogenous benzoic acid. Herein, we investigated the effects of benzoic acid on the variations of metabolome and proteome signatures in fermented goat milk *via* integrative metabolomics (LOQ 2.39–98.98 μg *L*^−1^) and proteomics approach based on UHPLC-Q-Orbitrap HRMS. Explicitly, benzoic acid reduced the content of taurine (7.06–4.80 mg *L*^−1^) and hypotaurine (3.86–1.74 mg *L*^−1^) due to a significant decrease in the levels of *glutamate decarboxylase 1* by benzoic acid. The reduction in lactose (7.13–5.31 mg *L*^−1^) and d-galactose (4.39–3.37 mg *L*^−1^) content was related to the decrease in *α-lactalbumin* and *β-galactosidase* levels, respectively, in fermented goat milk containing 40.00 mg *L*^−1^ benzoic acid. Meanwhile, the levels of maltose (22.84–16.53 mg *L*^−1^) and raffinose (4.19–3.10 mg *L*^−1^) progressively decreased with increasing benzoic acid concentrations (0.00–40.00 mg *L*^−1^), which had detrimental effects on the nutritional quality of fermented goat milk. Additionally, the concentration of benzoic acid and fermentation temperature are the most important factors to control the loss of nutrients in fermented dairy products.

## Highlights

• Benzoic acid as hydrions provider altered the β-galactosidase enzymes signatures.
• Benzoic acid caused the nutritional deterioration of fermented goat milk quality.
• Sulfonic acid derivatives and carbohydrates characterization were interfered.
• 142 critical metabolites were found in fermented goat milk treated with benzoic acid.
• Human health risk to benzoic acid is assessed by fermented dairy products.

## Graphical abstract

**Figure:**
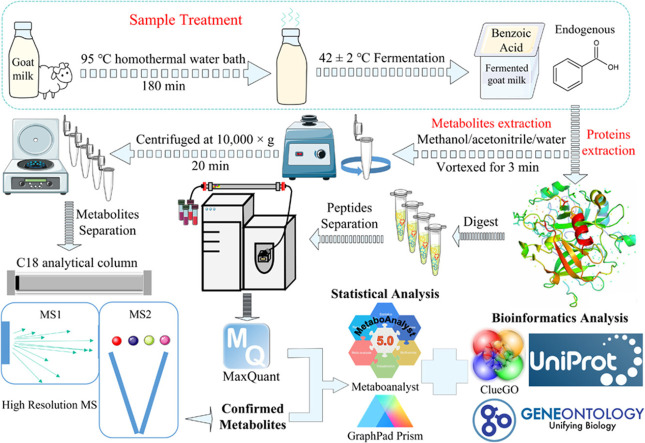

## 1. Introduction

Antimicrobial and antifungal agents (benzoic acid: C_6_H_5_COOH) have been widely used in the field of food safety for many years to inhibit the spoilage of yeasts, bacteria, and molds [1]. As an example, benzoic acid is broadly applied in the sterilization of beverages [2]. Nevertheless, benzoic acid is a common chemical and food preservative that is prohibited in dairy products. Available evidence showed that 76.8% of milk samples (109 samples tested) contained benzoic acid, ranging from 0.51 to 111 mg kg^−1^ [1], [3]. An extensive review of the toxicity of benzoic acid has suggested that benzoic acid interferes with intermediary metabolisms, including fatty acid metabolism, the tricarboxylic acid cycle, and the urea cycle [1]. However, benzoic acid formed by lactic acid bacteria and synthesized by the microbial enzymatic conversion of hippuric acid was naturally found in cheese [4], [5], [6] and fermented dairy products (Table S1, Electronic Supplementary Material) [7], [8], [9], [10], [11], [12], [13]. In addition, two mechanisms for the formation of benzoic acid found in fermented goat milk originate from the enzymatic degradation and microbial breakdown of phenylvaleric acid and phenylalanine, and the auto-oxidation of benzaldehyde [9,12]. Low doses of benzoic acid added to dairy products have been observed to have adverse health effects in infants and sensitive individuals, such as urticaria, convulsions, asthma, and metabolic acidosis [1].

Compared with cow milk, goat milk is less inflammatory, more easily digestible, and contains fewer allergens, making it superior in terms of its effects on the physiological functions of humans [13], [14]. At present, the industrial processing of goat milk requires improvement. The global dairy industry is profiting from technological innovations that enable it to provide high-quality services and products, thus becoming a significant contributor to the economy [15]. Within the dairy sector, yogurt (a fermented dairy product) has been perceived as a healthy food by consumers for centuries owing to the presence of bioactive components (lipoproteins, free amino acids, peptides, oligosaccharides, etc.) generated during the fermentation process [16]. Fermented goat milk is an excellent carrier for probiotics, as defined clearly by the Food and Agriculture Organization of the United Nations/World Health Organization: “living microorganisms which when administered in adequate amounts confer a health benefit on the host” [17]. To date, numerous investigations have focused on the formation, detection, and analysis of benzoic acid in fermented dairy products (yogurt and cheese), together with the nutritional value and sensory quality of fermented dairy products, but few such studies have considered the toxicity of benzoic acid in fermented dairy products, as it may affect the quality and nutritional value of fermented goat milk, thereby affecting nutrients levels [1,8,10,12,[18], [19]]. Several researchers have emphasized that the maximum admissible limit for benzoic acid in fermented dairy products is 40.0 mg kg^−1^ [18]. In accordance with data mining, benzoic acid (0.00–40.00 mg *L*^−1^) was detected in sixty fermented goat milk samples in six replicates, indicating the existence of endogenous benzoic acid. Metabolomics methodology is a high-throughput approach that aims to determine the effects of endogenous benzoic acid on the whole metabolic profile of fermented dairy products and can be used to detect biologically active endogenous metabolites [7,20]. Through the identification and annotation of a vast diversity of metabolites, the key metabolic pathways can be concentrated, the metabolic networks can be established, and the function of metabolites can be speculated. Proteomics is a fundamental technique to systematically identify and characterize the activity, function, structure, quantity, and molecular interactions of differentially expressed proteins (i.e., enzymes) [20,21]. Therefore, proteomics possesses potential applications to study the stimulation of endogenous benzoic acid in fermented goat milk at the whole-proteome level. To date, few investigations have reported the spatial proteomic and metabolomic profiles of fermented goat milk containing endogenous benzoic acid based on ultra-high performance liquid chromatography coupled to hybrid quadrupole-Orbitrap high-resolution mass spectrometry (UHPLC-Q-Orbitrap HRMS).

This study aims to systematically elucidate the relationship between metabolite-enzyme interactions and nutritional quality to improve the understanding of the nutritional quality of fermented goat milk containing endogenous benzoic acid. An integrated metabolomics and proteomics method based on UHPLC-Q-Orbitrap HRMS was applied to dissect the biological processes and metabolic pathways involved in metabolites and enzymes in fermented goat milk containing six final concentrations of benzoic acid. This information will be conducive to assessing the safety of fermented goat milk containing endogenous benzoic acid for further understanding the interactions between benzoic acid and its endogenous metabolites and enzymes. Additionally, the concentration of benzoic acid and fermentation temperature are the most important factors to control the loss of nutrients in fermented dairy products.

## 2 Materials and methods

### 2.1 Chemicals and reagents

UHPLC-grade acetonitrile, methanol, and formic acid (≥ 95%) were obtained from Thermo Fisher Scientific (Waltham, MA, USA). UHPLC-grade ammonium formate (≥ 99.9%) was purchased from Fluka (Chemie AG, Buchs, Switzerland). Ultrapure water (18.2 MΩ·cm) was generated by a Milli-Q Plus Water Purification System (Bedford, MA, USA). Fermented dairy product starter cultures containing *Lactobacillus delbrueckii* subsp. *Bulgaricus* and *Streptococcus thermophilus* were provided by Angel Yeast Co., Ltd. (Yichang, Hubei, China). All metabolites standards served as qualitative and quantitative reference compounds, including the following twenty-six authentic analytical standards: d-galactose, d-glucose, lactose, maltose, raffinose, UDP-galactose, ethyl undecylenate, glycerol triricinoleate, octyl isocyanate, trioctyl citrate, γ-linolenic acid ethyl ester, adenosine 3′-phosphoric acid, glucose 1-phosphate, hippuric acid, palmitic acid, stearic acid, octylamine, oleoylethanolamide, spermidine, hypotaurine, taurine, octadecylacrylamide, oleamide, ziziphin, robinetin, and hypoxanthine were supplied by Sigma-Aldrich (St. Louis, MO, USA) and Aladdin Bio-Chem Technology Co., Ltd. (Shanghai, China).

### 2.2 Samples preparation

Benzoic acid (0.00–40.00 mg *L*^−1^) was detected from sixty fermented goat milk samples in six replicates, indicating the existence of endogenous benzoic acid. Several researchers emphasized that the maximum admissible limit for benzoic acid in fermented dairy products is 40.0 mg kg^−1^ [18]. Sixty brands of whole goat milk samples were obtained from local stores in Shaanxi Province, Northwest China. Sixty whole goat milk samples were randomly divided into twelve groups, with five samples per group, and pooled. Twelve blends of goat milk were filtered and distributed into five groups, making a total of sixty samples. Fermented goat milk samples were prepared based on published investigations [22]. Goat milk samples were heated to 95 °C for 180 min in a thermostatic water bath followed by cooling to 42–45 °C (the ideal temperature for adding starter cultures). At 42 ± 2 °C, yogurt starter cultures containing *Lactobacillus delbrueckii* subsp. *Bulgaricus* and *Streptococcus thermophilus* were added to goat milk at a concentration of 10^6^ CFU/mL in a thermostatic water bath until the pH was 4.5 ± 0.1. Endogenous benzoic acid (0.00–40.00 mg *L*^−1^) was detected from sixty fermented goat milk samples. The final variable concentrations of endogenous benzoic acid in fermented goat milk were 0 mg *L*^−1^, 5 mg *L*^−1^, 10 mg *L*^−1^, 20 mg *L*^−1^, 30 mg *L*^−1^, and 40 mg *L*^−1^, respectively. The whole experiment was conducted in six replicates, and samples from each experiment were analyzed in triplicate (*n* = 18; 3 × 6).

### 2.3 Metabolomics analysis

Metabolite extraction was performed according to a previously validated protocol with minor modifications [22]. Each fermented goat milk sample (2 mL) containing benzoic acid with final variable concentrations of 0 mg *L*^−1^, 5 mg *L*^−1^, 10 mg *L*^−1^, 20 mg *L*^−1^, 30 mg *L*^−1^, and 40 mg *L*^−1^ was extracted in precooled methanol/acetonitrile/water solvent (5 mL: 2:2:1). The mixture was vortexed for 3 min and treated with ultrasonic for 10 min in an ice bath. Afterwards, the mixed samples were centrifuged (4 °C) at 10,000 *× g* for 20 min. Finally, the supernatant was collected and filtered with a 0.22-μm microporous membrane before liquid chromatography separation. Quality control (QC) samples were also prepared by mixing the extract of each sample. A series of blanks, QC samples, and other samples were analyzed to access the repeatability and stability of the instrument throughout the whole analysis.

Metabolite separation was conducted by Ultimate 3000 UHPLC system (Thermo Fisher Scientific) equipped with a Hypersil Gold column (C18, 2.1 mm i.d. × 100 mm, 5 μm, Thermo Fisher Scientific) at 300 μL min^−1^ and 35 °C. The injection volume was 5 μL for a total run time of 15 min. The mobile phase contains (A) water diluted with 0.1% formic acid containing 4 mM ammonium formate and (B) acetonitrile diluted with 0.1% formic acid containing 4 mM ammonium formate. Elution gradients were as follows: 12–95% B at 0–8 min, 95–100% B at 8–9 min, 100% B at 9–11 min, 100–12% B at 11–11.1 min, 12% B at 11.1–15 min. The heated electrospray ionization source (HESI) operated in negative- and positive-ion modes. MS acquisition parameters of the instrument were as follows: sheath gas, 35 arbitrary units; auxiliary gas, 10 arbitrary units; vaporizer temperature, 350 °C; spray voltage, 3.5 kV; auxiliary gas heater temperature, 320 °C. Nitrogen acts as a drying gas and nebulizer. Full scan MS (*m/z* 100–1500) was acquired in Q-Orbitrap with a resolution of 70,000 full-width at half of the maximum (*FWHM*), and an automatic gain control (AGC) target at 1.0 × 10^6^. The resolution of data-dependent secondary scan (dd-MS2) acquisition mode was 35,000 *FWHM*. The high energy collision-induced dissociation (HCD) was set to step modes (17.5 eV, 35 eV, 52.5 eV), and an AGC target was 2.0 × 10^5^.

### 2.4 Proteomics analysis

To determine the effect of benzoic acid on proteins of fermented goat milk, the sample preparation was modified as the procedure previously reported [23]. Briefly, 15 mL fermented goat milk containing benzoic acid with concentrations of 0 mg *L*^−1^ and 40 mg *L*^−1^ were mixed with 4% sodium dodecyl sulfate, 100 mM Tris–HCl, 1 mM dithiothreitol and a pH 7.6 buffer, and then boiled for 15 min. The extract was centrifuged at 4 °C, for 40 min at 14,000 *× g.* The supernatant was aspirated, and the extracted protein concentrations were assayed by the Bicinchoninic Acid Assay protein detection kit (Thermo Fisher Scientific). In short, samples were first reduced by dithiothreitol (56 °C for 1 h) with the final concentration of 10 mM. Afterwards, the mixture was cooled to room temperature and alkylated by adding the final concentration of iodoacetamide (55 mM) in darkness at 25 °C. After incubation for 30 min in darkness, the solution was transferred into 10 kDa ultrafiltration units (Merck, Darmstadt, Germany) and centrifuged for 15 min at 14,000 *× g.* With discarding the eluate, the filter was washed with UA buffer (8 M urea, 150 mM Tris–HCl, pH 8.0), followed by centrifugation for 15 min at 14,000 × *g* and repeated two times. After that, the filter was washed two times with 50 mM NH_4_HCO_3_ buffer to remove excess detergent, iodoacetamide, and dithiothreitol, and then centrifuged with 14,000 × *g* for 15 min. Finally, the remaining protein suspension was digested in 50 mM NH_4_HCO_3_ buffer for 16 h at 37 °C with 0.4 mg mL^−1^ trypsin (trypsin: protein = 1:50, *w/w*). The obtained peptides were acidified with 1% formic acid (100 μL) to stop the digestion. The resulting peptides were centrifuged at 14,000 *× g* for 10 min, collected as filtrate, and then desalted by C18 cartridges (Millipore Corporation, Bedford, MA, USA). All centrifugation sequences were executed at 4 °C. These samples were filtered with a 0.22-μm polyvinylidene fluoride membrane and analyzed by UHPLC-Q-Orbitrap HRMS.

The peptide mixture (20 μL) was injected into a C18 reversed-phase separation column (20 mm i.d. × 100 mm, 5 μm) and loaded on a C18 analysis column (75 μm i.d. × 150 mm, 3 μm, Thermo Fisher Scientific). The peptide mixture was separated by buffer solution A, 0.1% formic acid in ultrapure water, and buffer solution B composed of 0.1% formic acid in 80% acetonitrile using an Ultimate 3000 nano HPLC system (Thermo Fisher Scientific). The flow rate was 300 nL min^−1^ with a 100 min gradient. The linear gradient program of the mobile phase was applied as follows: 0–50 min, 5%–35% B; 50–75 min, 35%–100% B; 75–90 min, 100% B; 90–90.1 min, 100%–5% B; 90.1–100 min, 5% B. All identified peptide fragments and tandem mass spectrometry data were collected in positive-ion mode, operated in full scan MS with a selected mass range of *m/z* 300–1800 (an AGC of 1.0 × 10^6^ and a resolution of 70,000 *FWHM*), followed by ddMS2 acquisition method. Top 20 precursor ions were selected for ddMS2 acquisition using HCD fragmentation with a resolution of 35,000 *FWHM*. The AGC target for ddMS2 analysis was set to 2.0 × 10^5^.

### 2.5 Statistical analysis

The UHPLC-Q-Orbitrap HRMS raw data of untargeted metabolomics were exported from Xcalibur™ 4.1 software (Thermo Fisher Scientific). Extract all chromatographic peaks conforming to the set criteria: the signal-to-noise ratio of molecular species was 3, the minimum peak intensity was 1.0 × 10^7^, and the chromatographic peak was Gaussian graphical model. After selecting retention time (RT), accurate mass, and MS/MS fragments of each adduct by mass migration, comparing MS1 and MS2 data with spectral and accurate mass databases to obtain a list of metabolites containing compound names, molecular formulas, RTs, and accurate masses. Metabolites with relative standard deviations (RSD) less than 6.33% in test and QC samples were imported into MetaboAnalyst 5.0 for multivariate statistical analyses, such as supervised partial least squares discriminant analysis (PLS-DA) and unsupervised principal component analysis (PCA). At the same time, the clustering of samples and the relative abundance of metabolites were revealed by a heatmap.

MaxQuant version 1.6.17.0 (Max Planck Institute of Biochemistry, Germany) was employed to analyze the acquired raw file. The identification parameters were as follows: Uniport *capra hircus* database; maximum deletion cleavage, 2; trypsin as digestion enzyme; first search, 20 ppm; MS/MS tolerance, 20 ppm; main search tolerance, 6 ppm; maximum missed cleavage, 2; minimum peptide length, 7; fixed modifications, carbamidomethyl (C); label-free quantification, LFQ; variable modification, oxidation (M) and acetyl (protein N-term); decoy mode, revert. The false discovery rate was set to 0.01 for both proteins and peptides. To profile the function of the differentially abundant proteins, Gene Ontology (GO) enrichment analysis was performed by the GO Resource. KEGG pathway enrichment was visualized and executed by inputting the differentially abundant proteins highlighted by simultaneously considering *p*-value < 0.05, VIP scores > 1, and the cut-off value of fold change > 2 or < 0.5 to App ClueGO of Cytoscape version 3.8.2, in which nodes represent differentially abundant proteins and their KEGG pathways.

## 3 Results and discussion

### 3.1 Untargeted metabolic analysis of fermented goat milk containing endogenous benzoic acid

**Fig. 1:**
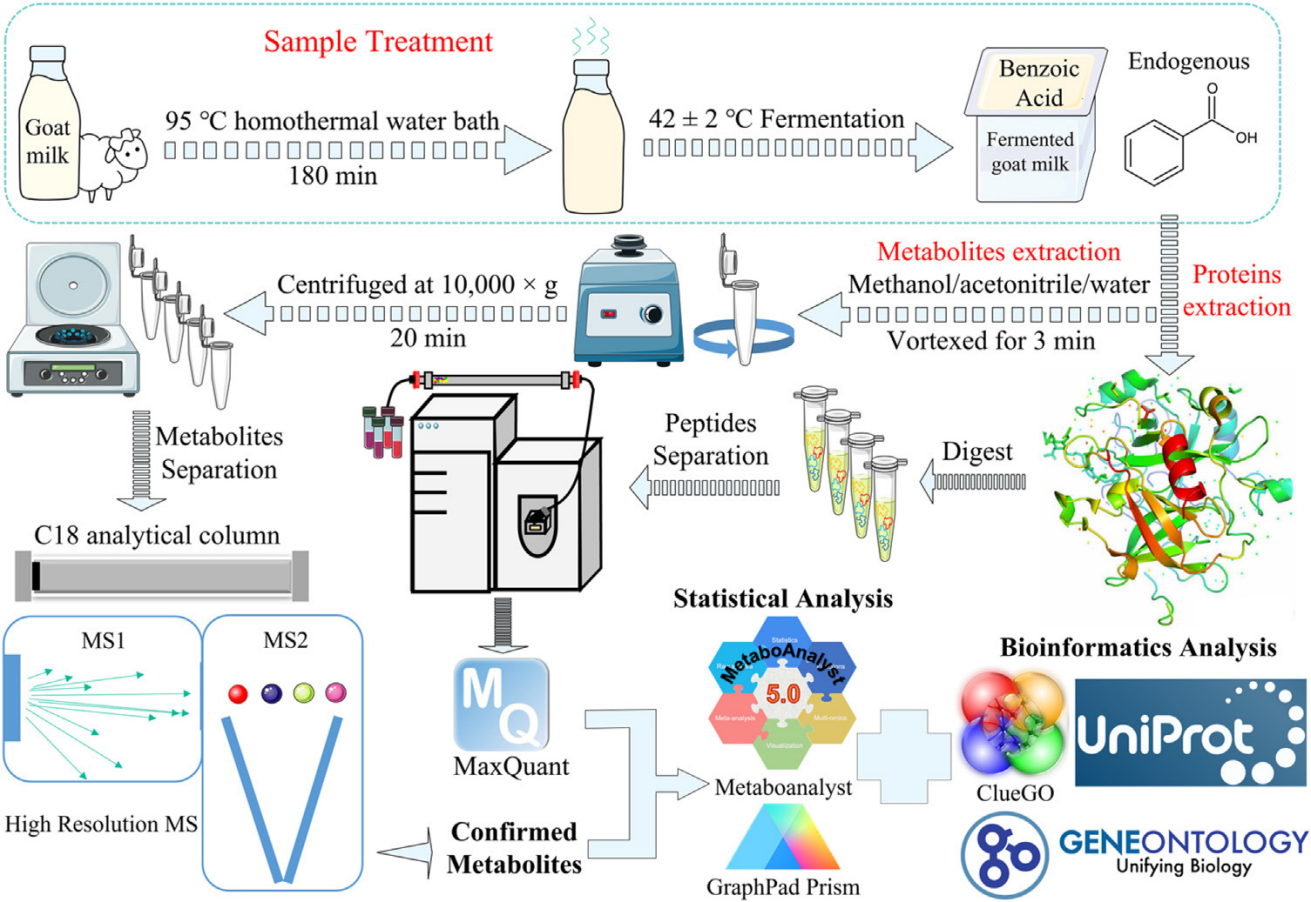
**The diagrammatic sketch of metabolites and proteins analysis was based on mass spectrometry-driven untargeted metabolomics and proteomics**.

**Fig. 2:**
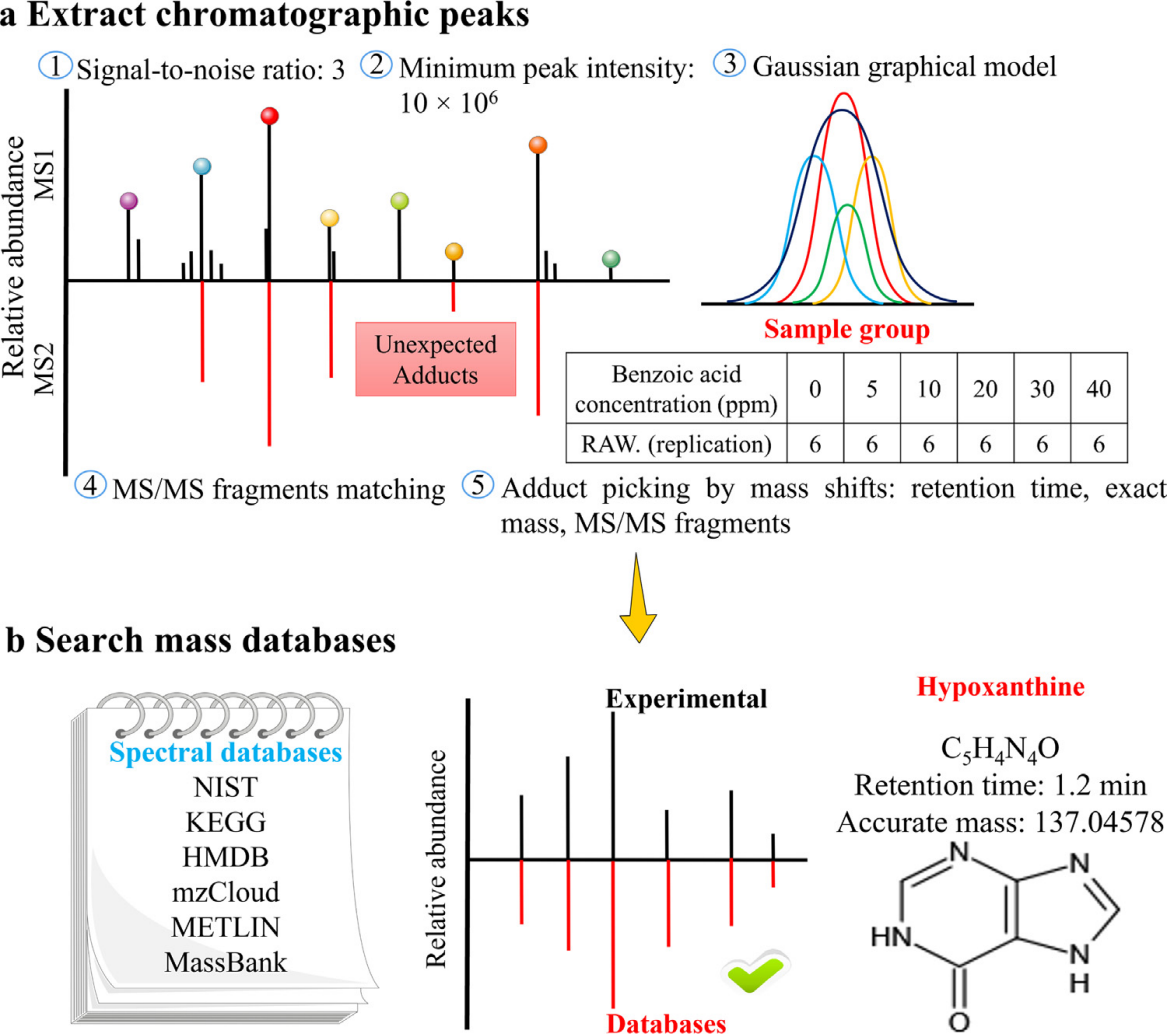
**Metabolites annotated signpost in fermented goat milk with six final benzoic acid concentrations.** (a) Extraction of all chromatographic peaks conforming to five set criteria. (b) Mass spectral and accurate mass databases were utilized to complete a tentative identification and annotation of metabolites.

Untargeted metabolomics measurements were carried out by UHPLC-Q-Orbitrap HRMS (theoretically, no limitations in the number of monitoring potential metabolites) devoted to analyzing the accrued metabolic variations in fermented goat milk at six final concentrations (benzoic acid C_7_H_6_O_2_: 0.00–40.00 mg *L*^−1^). Notably, quadrupole-Orbitrap HRMS has an ultrahigh-field orbitrap analyzer, and a high-performance quadrupole with the advantages of high mass precision, large charge capacity, wide dynamic range, high mass resolution, and continuously high spectral quality for sensitive detection. This helps to resolve target ions in complex samples from various possible interference and chemical background. The signpost (Figs. 1 and 2) for metabolite discoveries aims to: (*i*) After applying adequate preprocessing, full scan/data-dependent secondary scan (Full MS/dd-MS2) acquisition modes were used to acquire the chromatographic information and composite mass spectra (i.e., a combination of high-resolution MS and MS/MS spectrum) of all potential compounds in fermented goat milk. False-positive peaks and their spectra considered to be different adduct types, isotopic ions, and other background ions should be excluded before annotating analytes. Then, all chromatographic peaks conforming to the set criteria were extracted (Fig. 2a). *(ii)* After picking up RT, accurate mass, and MS/MS fragments of adducts by mass migration, structure elucidation was performed to compare MS1 and MS2 data with NIST, KEGG, HMDB, online mzCloud, and METLIN databases. A list of compound names, molecular formulas, RTs, and accurate masses were obtained after providing several matches with mass errors below 3.28 ppm and matching with the accurate mass databases of the authentic analytical standards, thus completing a tentative identification and annotation of metabolites (Fig. 2b). A total of 8356 ion peaks were acquired. *(iii)* Mass Frontier 8.0′s HighChem Fragmentation Library™ combined with exclusive Fragment Ion Search (FISh), metabolites were recharacterized. Mass Frontier 8.0 exploits the HighChem Fragmentation Library™ to rapidly and accurately allocate the secondary fragmentation structures of metabolites. Then, FISh organically combines with metabolic pathways and shared fragments of parent ions and product ions to capture comprehensive chemical insights and fragmentation pathways of metabolites towards biological processes.

**Fig. 3:**
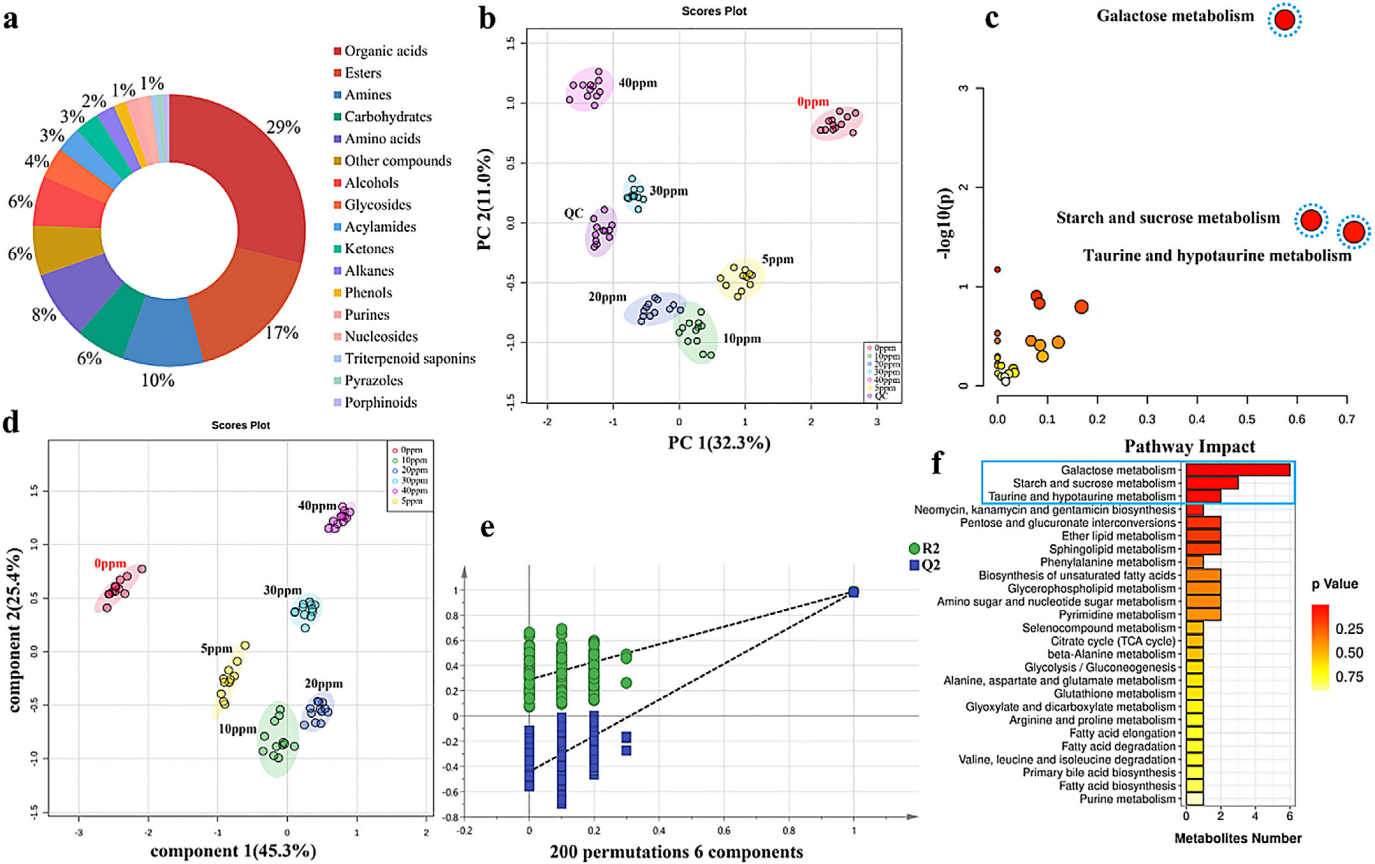
**Multivariate statistical analysis of identified metabolites in fermented goat milk with six final benzoic acid concentrations.** (a) Distribution of identified metabolites in fermented goat milk with six final benzoic acid concentrations. (b) PCA score plot of the differential metabolites. (c) Metabolomics view map of metabolic pathways in fermented goat milk. (d) PLS-DA score plot of the differential metabolites. (e) Validation of PLS-DA model by 200 permutation tests. (f) Pathway enrichment analysis of differential metabolites. Class relates to sample groups and 0 ppm, 5 ppm, 10 ppm, 20 ppm, 30 ppm, and 40 ppm represent fermented goat milk with 0 mg *L*^−1^, 5 mg *L*^−1^, 10 mg *L*^−1^, 20 mg *L*^−1^, 30 mg *L*^−1^, and 40 mg *L*^−1^ benzoic acid, respectively. The *p*-values in heatmap range from 0.25 (red) to 0.75 (yellow).

As an example, by selecting RT, accurate mass, and MS/MS fragments of the sample peak, as well as comparing them with the databases, a metabolite named hypoxanthine was obtained. Its molecular formula is C_5_H_4_N_4_O, its mass-to-charge ratio (*m/z*) is 137.04578, its RT is 1.2 min, and its structure was depicted in Fig. 2b. The chromatographic peak (*m/z* 137.04578) matching the set criteria was extracted from the sample to ensure that it contains hypoxanthine metabolite. To rule out the possibility of isomers, *m/z* 137.04578 was confirmed as metabolite hypoxanthine by comparing the experimental fragmentation spectral information of the sample with that of authentic analytical standard. Furthermore, the secondary fragment structures of the metabolite assigned by HighChem Fragmentation Library™ and the fragment fragmentation pathway captured by FISh reconfirmed the hypoxanthine metabolite. Based on this identification method, 135 differential metabolites were characterized in fermented goat milk with six final concentrations of benzoic acid, as listed in Table S2. Fig. 3a illustrated the classification of differential metabolites in the investigated fermented goat milk.

The untargeted metabolomics method was thoroughly validated from such aspects of recovery, precision, limits of detection (LOD) and quantification (LOQ), and linearity (Table S3). LOD and LOQ values were determined as the minimum spiked concentrations of the analytes and could be identified and quantified with acceptable authenticity, evaluated with three-fold and ten-fold signal-to-noise ratio, respectively. In the current trial, LOD and LOQ values for each analyte varied from 0.98 to 34.65 μg *L*^−1^ and 2.39–98.98 μg *L*^−1^, respectively. It demonstrated that the method had the high-test sensitivity for the determination and quantification of metabolites in fermented goat milk with different final concentrations of benzoic acid, as shown in Table S4 [6,16,[24], [25], [26], [27]]. The results of linear evaluation indicated that the determination regression coefficients (R^2^) of all external standards (ESs) (*p*-value < 0.05) were in the range of 0.9979 to 0.9999, indicating sufficient linearity of the method. The relative standard deviations (RSD) of ES peak areas in each metabolite were less than 6.33%, demonstrating the repeatability and reliability of the method. Moreover, the acceptable recoveries of all metabolites ranged from 90.22% to 108.91%, which were acceptable for the determination of metabolites in fermented goat milk with different final concentrations of benzoic acid. Ten representative metabolites with VIP scores (> 1) and high response (> 1 × 10^7^) including hypotaurine, taurine, d-galactose, lactose, UDP-galactose, glucose 1-phosphate, d-glucose, maltose, raffinose, and hippuric acid were selected for quantitative analysis by ESs quantitation method.

### 3.2 Disparate benzoic acid concentrations showed distinct metabolomic signatures

To further explore the metabolic alterations of fermented goat milk in response to the different benzoic acid concentrations, multivariate analyses such as PCA and PLS-DA were applied to identify the metabolites in fermented goat milk. The individual distribution of the six groups of fermented goat milk samples with different benzoic acid concentrations was clearly separated, implying significant variations in their metabolites (Fig. 3b). Major variations of the raw data explained by the first two principal components (PCs) were 32.3% and 11.0%, respectively. Meanwhile, PCA score plot displayed good overlapping and close clustering of QCs, illustrating the stability of the UHPLC-Q-Orbitrap platform and the reliability of the experimental method. As seen in Fig. 3d, the score plot of PLS-DA revealed a total variance of 70.7%, of which component 1 was 45.3% and component 2 was 25.4%. Fermented goat milk samples containing six disparate benzoic acid concentrations (0.00–40.00 mg *L*^−1^) were isolated and the results were consistent with those of PCA. In 200 permutation tests, the intercepts of R^2^ and Q^2^ were (0, 0.289) and (0, −0.442), respectively (Fig. 3e), which proved the superior goodness-of-fit and reliability of the PLS-DA model constructed from the mass spectrometry data of fermented goat milk. Variable importance in PLS-DA projection (VIP) scores were calculated and examined for each component. In this study, VIP scores > 1 and *p*-values < 0.05 (ANOVA) were regarded as thresholds to demonstrate the differential metabolites, and a total of 135 critical metabolites responsible for metabolic variations among six benzoic acid concentrations were determined.

**Fig. 4:**
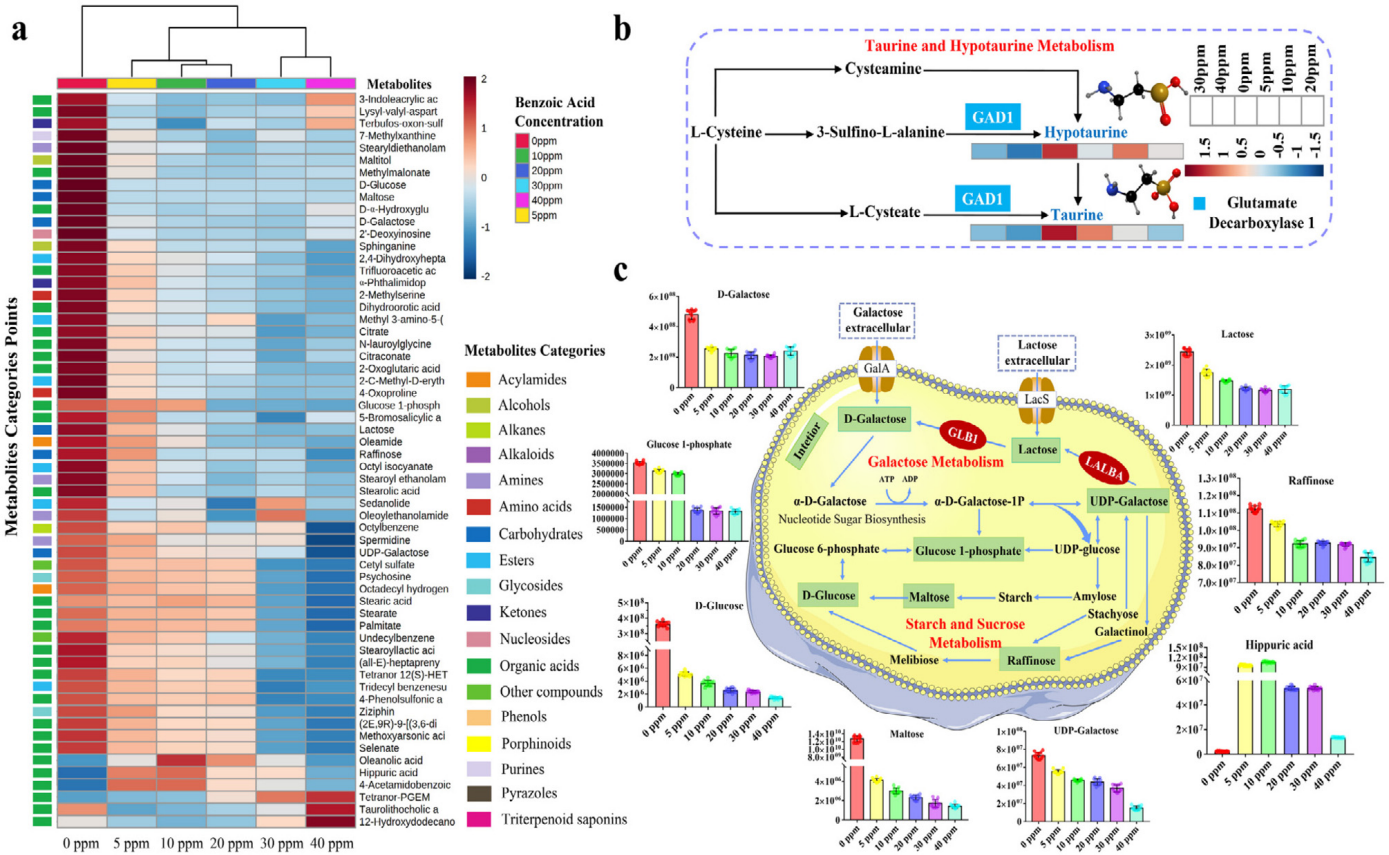
**The strategy of untargeted metabolomics for metabolites and KEGG pathway analysis.** (a) Heatmap of fifty differential metabolites. The blue color indicated low, and the red color indicated high. Class related to 0 ppm, 5 ppm, 10 ppm, 20 ppm, 30 ppm, and 40 ppm represent fermented goat milk with 0 mg *L*^−1^, 5 mg *L*^−1^, 10 mg *L*^−1^, 20 mg *L*^−1^, 30 mg *L*^−1^, and 40 mg *L*^−1^ benzoic acid, respectively. Scales with the range from –2 (blue) to 2 (red) represent the trend of metabolites’ peak area after logarithmic conversion. (b) Schematic of the taurine and hypotaurine metabolism pathway, and characterization of peak areas of two critical metabolites (hypotaurine and taurine) in fermented goat milk with six final benzoic acid concentrations. (c) Schematic of the galactose metabolism, starch and sucrose metabolism pathways, and characterization of peak areas of eight critical metabolites in fermented goat milk with six final benzoic acid concentrations. Metabolites of black words were undetected and insignificantly changed in samples. The remaining representatives were detected and significantly changed in samples. The columns and rows were ordered based on hierarchical clustering. GAD1 = *glutamate decarboxylase 1*, LALBA = *α-lactalbumin*, GLB1 = *β-galactosidase*.

As a more rigorous test, unsupervised hierarchical clustering was performed for the selected metabolites to assess the accuracy and plausibility of diverse biological datasets, and to compare the dynamic changes of these differential metabolites. As depicted in Fig. 4a, the clustering analysis based on average algorithm proved that the samples can be divided into three clusters. Cluster I included 0 mg *L*^−1^, cluster II included 5 mg *L*^−1^, 10 mg *L*^−1^, and 20 mg *L*^−1^, while 30 mg *L*^−1^ and 40 mg *L*^−1^ in cluster III, which was highly in accordance with the results of PCA model.

### 3.3 Transformation pathway analysis of characteristic metabolites

To elucidate alterations in metabolic pathways of metabolites in fermented goat milk with six final benzoic acid concentrations, 135 differentially endogenous and exogenous metabolites were submitted to the KEGG database for pathway topology and enrichment analysis. The results showed that sixty-two metabolites mapped to metabolism and possessed KEGG IDs. Topology analysis with –log(P) was displayed in Fig. 3c, and the *p*-value can be applied to predict significantly correlated pathways. Sixty-two differentially abundant metabolites were enriched in twenty-seven KEGG pathways. By calculating the *p*-value and impact value of each pathway, galactose metabolism (2.05E-04, *p*-value; 0.58, impact value) was the most enriched pathway, and starch and sucrose metabolism (2.14E-02, *p*-value; 0.63, impact value) and taurine and hypotaurine metabolism (2.82E-02, *p*-value; 0.71, impact value) were also the main enriched pathways (Fig. 3c and 3f). Therefore, according to the variations of endogenous and exogenous metabolites in fermented goat milk and the enrichment pathways reported in the KEGG database, there were three major pathways (Fig. 4b, taurine and hypotaurine metabolism and Fig. 4c, galactose metabolism, starch and sucrose metabolism) in fermented goat milk with six benzoic acid concentrations.

### 3.4 Protein expression profiling of fermented goat milk containing endogenous benzoic acid

**Fig. 5:**
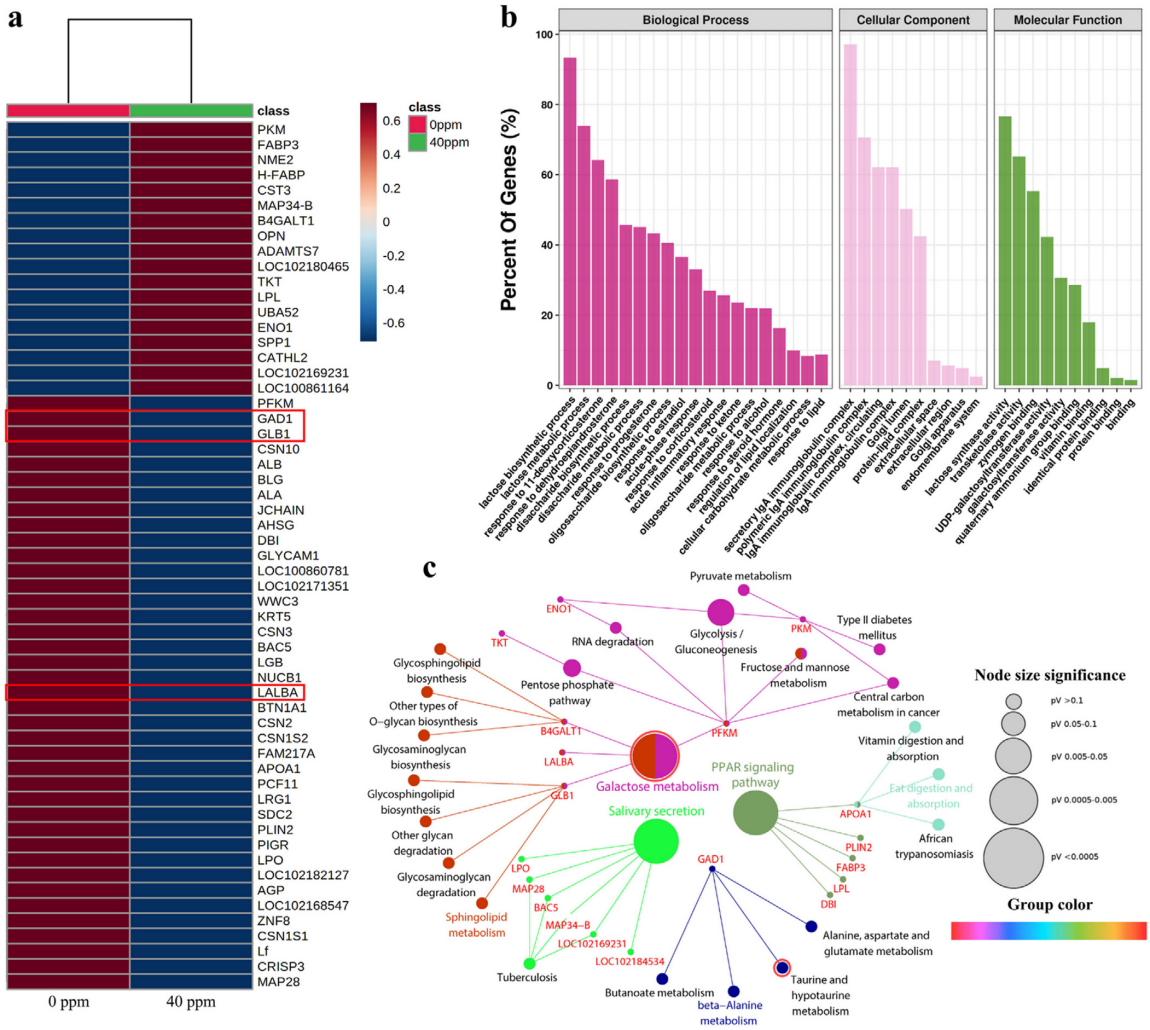
**Comparative proteomics analysis of fermented goat milk with two final benzoic acid concentrations (0.00 mg*****L***^**−1**^**and 40.00 mg*****L***^**−1**^**).** (a) Heatmap visualization of fifty-seven differentially expressed proteins. The blue color indicated low, and the red color indicated high. (b) Gene Ontology classification of differentially expressed proteins. (c) ClueGo visualization of pathway enrichment analysis obtained after functional classification analysis of differentially expressed proteins. Nodes represent differentially abundant proteins and their KEGG pathways. The size of the nodes ranges from little (less significant; pV > 0.05) to big (highly significant; pV < 0.0005).

To explore the potential mechanisms of benzoic acid in fermented goat milk, the protein expression of fermented goat milk containing 0 mg *L*^−1^ and 40 mg *L*^−1^ benzoic acid was compared. In total, 167 proteins were identified in fermented goat milk, and fifty-seven differentially abundant proteins were highlighted by simultaneously considering *p*-values < 0.05, VIP scores > 1 and the cut-off value of fold change > 2 or < 0.5, as displayed in Table S5. Unsupervised hierarchical cluster analysis was used to determine the differences in proteomic between fermented goat milk with final benzoic acid concentrations of 0 mg *L*^−1^ and 40 mg *L*^−1^. Fifty-seven significantly abundant proteins in the two sample groups were clustered, and the results were displayed in a heatmap (Fig. 5a) to obtain visual information of the data. To evaluate the major biological processes in fermented goat milk affected by benzoic acid, fifty-seven identified proteins were classified using GO annotation and further categorized into functional activities, i.e., biological process, molecular function, and cell component (Fig. 5b). According to the annotation of differentially expressed proteins, the most constantly occurring biological processes were lactose biosynthetic process (93.4%) and lactose metabolic process (73.9%), followed by disaccharide biosynthetic process (45.6%), disaccharide metabolic process (45.1%) and oligosaccharide biosynthetic process (40.5%). Molecular functions revealed that differentially expressed proteins played prominent roles in lactose synthase activity (76.6%), UDP-galactosyltransferase activity (42.3%) and galactosyltransferase activity (30.7%). Meanwhile, the most prevalent cellular components of the differentially expressed proteins were predominantly located in the Golgi lumen (50.2%) and extracellular space (7.0%). For the cellular components, fifty-seven differential proteins of the goat milk group were mainly distributed in the extracellular space and extracellular region [23]. KEGG pathway enrichment was executed to apply the Cytoscape plug-in ClueGO App, and twenty-five KEGG pathways of protein enrichment were presented in Fig. 5c. The differentially abundant proteins participated in crucial metabolic pathways related to galactose metabolism (GLB1, LALBA) and taurine and hypotaurine metabolism (GAD1). Crucial metabolic pathways, it should be noted, were consistent with the significant pathways of metabolomics.

### 3.5 Integrative metabolomics and proteomics analysis for interpretation of pathways

Sulfonic acid derivatives, such as taurine and hypotaurine (Fig. 4b), play essential roles in human health due to their protective properties for preventing neurodegenerative diseases [28], [29]. Taurine (2-aminoethanesulfonic acid) regarded as a “conditionally essential” amino acid in nutrition has several physiological functions, such as cell-volume modulation, antioxidative activity, membrane stabilization, mitochondrial protein translocation, intracellular calcium level regulation and the modulation of neuroendocrine function and nutrition [30]. Hypotaurine (2-aminoethanesulfinic acid), a cytoprotective agent and nonproteinogenic cysteine oxygenate, has a strong scavenging activity of active hydroxyl radicals as well as other health properties, such as hypocholesterolemic and antihypertensive properties [29,31]. Comparing the metabolites content in fermented goat milk containing six final concentrations of benzoic acid, we found that the metabolomic profiles of sulfonic acid derivatives (taurine, from 7.06 to 4.80 mg *L*^−1^; hypotaurine, from 3.86 to 1.74 mg *L*^−1^) decreased significantly (*p*-value < 0.05) with increasing benzoic acid concentrations (Fig. 4b). Irreversible decarboxylation of 3-sulfino-l-alanine and l-cysteate to hypotaurine and taurine, respectively, was efficiently catalyzed by *glutamate decarboxylase 1* (GAD1) (Fig. S2). By integrating proteomics and metabolomics data, increasing benzoic acid concentrations (from 0 to 40 mg *L*^−1^) reduced the level and activity of *glutamate decarboxylase 1* (Fig. 5a), which cannot efficiently catalyze the decarboxylation of 3-sulfino-l-alanine and l-cysteate to hypotaurine and taurine, respectively (Fig. S2). As an acidic environment (provided by benzoic acid) is crucial for *glutamate decarboxylase 1* activity [32], only a proton-rich environment is able to protonate l-cysteate^−^ and 3-sulfino-l-alanine^−^ (L-cysteate and 3-sulfino-l-alanine carry one negative charge, respectively) into l-cysteate^0^ and 3-sulfino-l-alanine^0^ (no net charge) and activate the GAD1 operon for transport, effectively catalyzing the irreversible decarboxylation of l-cysteate and 3-sulfino-l-alanine, respectively. The concentrations of hypotaurine and taurine were preliminarily confirmed to decrease with increasing benzoic acid concentrations. The explanation for the effect of benzoic acid (from 0 to 40 mg *L*^−1^) on taurine variations (from 7.06 to 4.80 mg *L*^−1^) can also be considered as follows: the oxygenation of hypotaurine to produce taurine, which decreased notably (*p*-value < 0.05) with increasing concentration of benzoic acid (Fig. 4b); the conjugation effect of taurine with benzoic acid (carboxylic acid) [33]. Therefore, sulfonic acid derivatives (taurine and hypotaurine) indirectly affected the nutritional quality of fermented goat milk.

Because of key carbohydrate utilization metabolisms, especially galactose metabolism and starch and sucrose metabolism, the dynamic variations of carbon catabolites in fermented goat milk were expounded (Fig. 4c). Beyond being the primary energy substances, digestible carbohydrates can have conducive effects on the health of the host, including as dietary fiber and prebiotics [34], [35]. Prebiotics, such as lactose and raffinose in fermented goat milk, can confer advantageous health benefits to the host since they stimulate the growth of favorable bacteria in the intestine microbiota and offer low-calorie products [36], [37]. Concerning metabolome data, the levels of fermentable carbohydrates providing nutritional value (e.g., d-galactose, UDP-galactose, d-glucose, lactose, maltose and raffinose) changed significantly (*p*-value < 0.05) in fermented goat milk with six final concentrations of benzoic acid. It was observed that the content of d-galactose, UDP-galactose, d-glucose, lactose, maltose and raffinose were lower in the benzoic acid treated groups (Fig. 4c), which was not conducive to energy supply and human demand. Meanwhile, d-glucose is a precursor for the synthesis of several nutrients (lactose, glycogen, cellulose, glycolipids, and glycoproteins), and can be decomposed into a variety of other biomolecules (galactose and lipids). d-glucose plays a key role in evaluating the nutritional quality of fermented goat milk with six final concentrations of benzoic acid [38]. It has been reported that d-galactose exists in free or bound form to other carbohydrate units in diverse glycosidic linkages, such as β−1,3, β−1,4 and α−1,6, and is a component of proteins and lipids in cells [39], [40]. In this study, the content of d-galactose (from 4.39 to 3.37 mg *L*^−1^), d-glucose (from 5.67 to 4.10 mg *L*^−1^), and lactose (from 7.13 to 5.31 mg *L*^−1^) decreased with increasing concentrations of benzoic acid in fermented goat milk (Fig. 4c). As the major carbohydrate component of milk, lactose is synthesized from UDP-galactose by *α-lactalbumin* (LALBA). It is well-known that the acidic pH provided by benzoic acid allows unrestricted hydrolysis of *α-lactalbumin*. In accordance with our results, the levels of *α-lactalbumin* decreased with increasing benzoic acid concentrations (0.00–40.00 mg *L*^−1^) (Fig. 5a). The biological value of *α-lactalbumin* decreased gradually with increasing concentration of benzoic acid [41]. In this research, *α-lactalbumin* (LALBA) could not efficiently synthesize lactose (from 7.13 to 5.31 mg *L*^−1^) (Fig. 4c). Additionally, *β-galactosidase* (*β-d-galactoside galactohydrolase*, E.C. 3.2.1.23, trivially lactase) is well-known to cleave the O-glycosylic bond in lactose and split it into d-galactose. The levels of *β-galactosidase* decreased with increasing benzoic acid concentrations (Fig. 5a), which can be considered as follows: the accumulation of hydrogen ions is indeed a factor that reduced *β-galactosidase* activity in fermented goat milk [42], [43]. In accordance with the results, the reduction in lactose (from 7.13 to 5.31 mg *L*^−1^) and d-galactose (from 4.39 to 3.37 mg *L*^−1^) levels were related to the decrease of *α-lactalbumin* (LALBA) and *β-galactosidase* (GLB1) levels in fermented goat milk containing 40 mg *L*^−1^ benzoic acid, respectively (Fig. 5a). Benzoic acid is metabolized to hippuric acid through *glycine-N-acylase*, while hippuric acid as the natural component of milk can be naturally converted to benzoic acid [44], [45]. As the transformation pathway, the content of hippuric acid (1.54 mg *L*^−1^) was very low at the concentration of 0 mg *L*^−1^ benzoic acid and then gradually decreased (from 34.39 to 5.53 mg *L*^−1^) with increasing benzoic acid concentrations (Fig. 4c). Disaccharide (maltose) could fill the part of the agent responsible for triggering signals that induce satiety and hunger in human beings [46]. The prebiotic potential of trisaccharide raffinose for human health is to increase the relative abundance of *Lactobacillus* and *Bifidobacterium* species, while reducing *Proteobacteria*, including well-known pathogens, such as *E. coli* [47], [48], [49]. The levels of maltose (from 22.84 to 16.53 mg *L*^−1^) and raffinose (from 4.19 to 3.10 mg *L*^−1^) decreased progressively with increasing benzoic acid concentrations, which had a detrimental effect on the nutritional quality of fermented goat milk (Fig. 4c).

### 3.6 Good manufacturing for controlling endogenous benzoic acid during goat milk fermentation

In fermentation by *Lactobacillus delbrueckii* subsp. *Bulgaricus* and *Streptococcus thermophilus*, the starter cultures for making fermented goat milk, benzoic acid (40.00 mg *L*^−1^) was highly detected at 40 °C (Fig. S1a), which had a detrimental effect on the nutritional quality of fermented goat milk. However, in fermentation by foregoing starter cultures for making fermented goat milk, the concentration of benzoic acid fluctuated between 0.89 and 2.12 mg *L*^−1^ at 4 °C (Fig. S1b). Additionally, in fermentation by foregoing starter cultures for making fermented goat milk, the average concentrations of lactose, taurine, hypotaurine, d-galactose, and maltose at 4 °C were close to those in fermented goat milk induced by 0 mg *L*^−1^ benzoic acid (Fig. S1c). Benzoic acid occurs naturally, and the amount produced varies depending on temperature. The results of this study could be used to establish the standard permission scope for benzoic acid in fermented dairy products and provide a future direction on how to control the degradation of the nutritional quality of fermented dairy products induced by benzoic acid. Together, ten differential metabolites and three pivotal enzymes related to the induction of benzoic acid in fermented goat milk, and the control of nutrients loss is also a critical factor. The explanation for the effect of benzoic acid on taurine formation can be considered as follows: the conjugation effect of taurine with benzoic acid (carboxylic acid); the oxygenation of hypotaurine to produce taurine, which decreased notably (*p*-value < 0.05) with increasing concentration of benzoic acid; and the level of *glutamate decarboxylase 1* decreased with benzoic acid concentration. Therefore, the concentration of benzoic acid and fermentation temperature are the most important factors to control the loss of nutrients in fermented dairy products.

## 4 Perspectives and conclusion

In general, metabolites and proteins changes in fermented goat milk containing six final concentrations of benzoic acid were investigated by untargeted metabolomics and quantitative proteomics approaches, while the latent effects of metabolites and proteins variations on nutritional quality were discussed. Proteomics helps bridge the connection between metabolomic and proteomic data and identify the pivotal enzymes responsible for these alterations. Untargeted metabolomics based on UHPLC-Q-Orbitrap HRMS is a powerful technology to describe the discrepancies in different benzoic acid concentrations, and it is also helpful to assess the dynamic variations of samples in response to different stimuli. In the current study, galactose metabolism, starch and sucrose metabolism, and taurine and hypotaurine metabolism were prominent metabolic pathways. Furthermore, ten differential metabolites, including d-galactose, UDP-galactose, d-glucose, lactose, maltose, raffinose, glucose 1-phosphate, hippuric acid, taurine and hypotaurine as well as three noteworthy enzymes, *α-lactalbumin, β-galactosidase* and *glutamate decarboxylase 1*, were related to the quality traits of fermented goat milk containing endogenous benzoic acid. Additionally, the concentration of benzoic acid and fermentation temperature are the most important factors in controlling the loss of nutrients in fermented dairy products. This study not only evaluated the chemical compositions, metabolic pathways and biological processes of metabolites and enzymes in fermented goat milk containing endogenous benzoic acid, but also provided more evidence for the future preservation and quality assessment of fermented goat milk. Overall, these findings offered a future direction for the investigation of the metabolic signatures of fermented goat milk containing endogenous benzoic acid.

## Declaration of competing interest

The authors declare that they have no conflicts of interest in this work.

## Appendix Supplementary materials

Image, application 1
