# Supplementary material for: Interference of endogenous benzoic acid with the signatures of sulfonic acid derivatives and carbohydrates in fermented dairy products
Source: Fundam Res. 2022 Nov 9;4(6):1523–32. doi: 10.1016/j.fmre.2022.09.033 (PMC11670729; doi:10.1016/j.fmre.2022.09.033)
Supplement: Supplementary file 1 [file mmc1.docx]

**Supporting Information for**

Interference of endogenous benzoic acid with the signatures of sulfonic acid derivatives and carbohydrates in fermented dairy products

Wei Jia^a, b, *^, Xin Wang ^a^, Lin Shi ^a^

^a^ *School of Food and Biological Engineering, Shaanxi University of Science & Technology, Xi'an 710021, China*

^b^ *Shaanxi Research Institute of Agricultural Products Processing Technology, Xi'an 710021, China*

**Table Captions**

**Table S1 Latest studies on the existence of benzoic acid in yogurts from cow, goat, and sheep milk.**

**Table S2 Differential compounds from fermented goat milk with six final benzoic acid concentrations (FDR-adjusted *p*-value ≤ 0.05).**

**Table S3 Summary of the method validation performance characteristics as determined for the untargeted metabolomics method.**

**Table S4 Comparison of the method performance (limits of detection and quantification) and determination of metabolites in the present work and the literatures.**

**Table S5 High-content differentially expressed proteins (FDR-adjusted *p*-value ≤  0.05) of fermented goat milk with two final benzoic acid concentrations (0.00 mg L^-1^ and 40.00 mg L^-1^).**

**Figure Captions**

**Fig. S1. Variations of endogenous benzoic acid during fermentation of goat milk.** (a) In fermentation by starter cultures for making fermented goat milk, benzoic acid fluctuated at the concentration of 40.00 mg L^-1^ at 40 °C. (b) In fermentation by starter cultures for making fermented goat milk, benzoic acid fluctuated at the concentration of 0.89 to 2.12 mg L^-1^ at 4 °C. (c) In fermentation by starter cultures for making fermented goat milk, the average concentration of lactose, taurine, hypotaurine, D-galactose, and maltose at 4 °C. The center line indicates the median, ****P < 0.0001; ns not significant using unpaired two-tailed Student’s t-test.

**Fig. S2. The chemical mechanism of benzoic acid interfering with two critical metabolites (taurine and hypotaurine).**


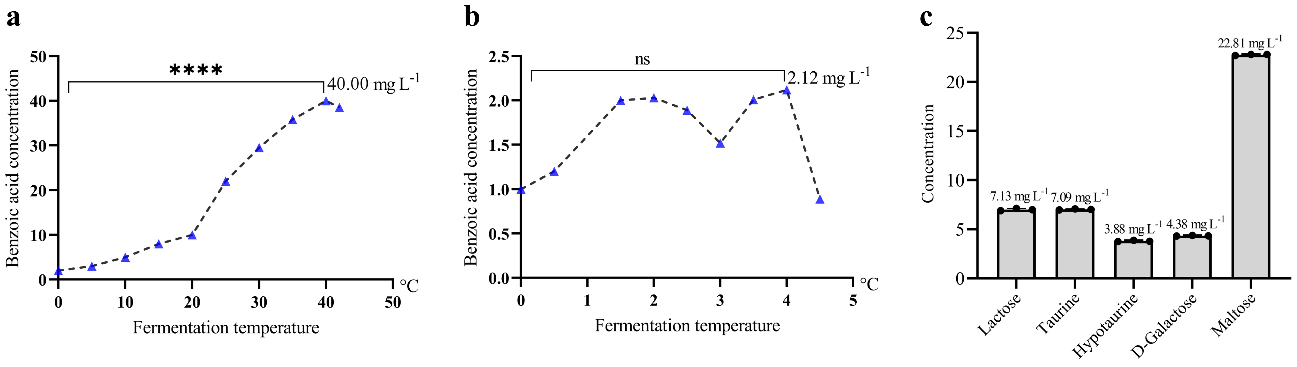


**Fig. S1. Variations of endogenous benzoic acid during fermentation of goat milk.** (a) In fermentation by starter cultures for making fermented goat milk, benzoic acid fluctuated at the concentration of 40.00 mg L^-1^ at 40 °C. (b) In fermentation by starter cultures for making fermented goat milk, benzoic acid fluctuated at the concentration of 0.89 to 2.12 mg L^-1^ at 4 °C. (c) In fermentation by starter cultures for making fermented goat milk, the average concentration of lactose, taurine, hypotaurine, D-galactose, and maltose at 4 °C. The center line indicates the median, ****P < 0.0001; ns not significant using unpaired two-tailed Student’s t-test.


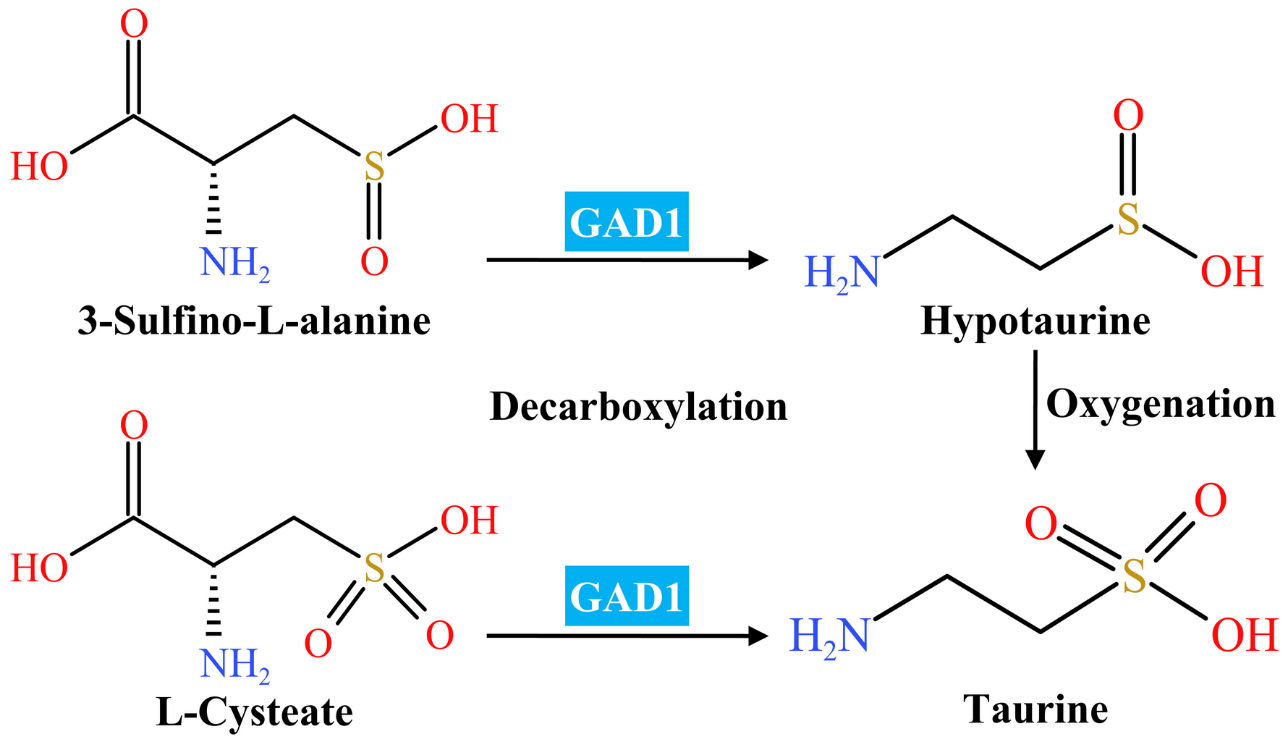


**Fig. S2.** **The chemical mechanism of benzoic acid interfering with two critical metabolites (taurine and hypotaurine).**

**Table S1 Latest studies on the existence of benzoic acid in yogurts from cow, goat, and sheep milk.**

| Sample category | Sample description | Positive rate (%) | Measurement method | Benzoic acid levels | Ref. |
| --- | --- | --- | --- | --- | --- |
| Yoghurts from  cow milk | Greek yogurt | 100 | UPLC-MS/MS ^a^ | 0-11.00 mg kg^-1^ | [7] |
|  | Cow milk yoghurt | 100 | UPLC/UV ^b^ | 5.29-20.72 mg kg^-1^ | [8] |
|  | Skim milk fermentation | 71.4 | HPLC-PDA ^c^ | 0-12.46 mg kg^-1^ | [9] |
|  | Skim milk fermentation | 100 | HPLC-PDA ^c^ | 0-14.55 mg kg^-1^ | [10] |
|  | Fermented cow milk | - | - | Up to 18.8 mg L^-1^ | [11] |
|  | Fermented milk | - | - | Up to 24 mg L^-1^ | [11] |
| Yoghurts from  goat milk | UHT goat milk kefir | 100 | HPLC-DAD ^d^ | 8.50-26.60 mg kg^-1^ | [12] |
|  | Goat milk yoghurt | 100 | UPLC/UV ^b^ | 5.43-30.72 mg kg^-1^ | [8] |
|  | Goat fermented milk | - | - | Up to 25 mg L^-1^ | [11] |
|  | Fermented goat milk | 93.8 | UHPLC-Q-Orbitrap HRMS ^e^ | 0.00-40.00 mg L^-1^ | Present work |
| Yoghurts from  sheep milk | Sheep milk yoghurt | 100 | UPLC/UV ^b^ | 31.60-54.34 mg kg^-1^ | [8] |
|  | Sheep fermented milk | - | - | Up to 36 mg L^-1^ | [11, 13] |

^a^ UPLC-MS/MS stands for ultra-high-performance liquid chromatography coupled with tandem mass spectrometry.

^b^ UPLC/UV stands for high performance liquid chromatograph with Ultraviolet Detector.

^c^ HPLC-PDA stands for high performance liquid chromatograph coupled with a photodiode array detector.

^d^ HPLC-DAD stands for high performance liquid chromatograph with diode array detector.

^e^ UHPLC-Q-Orbitrap HRMS stands for ultrahigh-performance liquid chromatography coupled to hybrid quadrupole-Orbitrap high-resolution mass spectrometry.

**Table S2 Differential compounds from fermented goat milk with six final benzoic acid concentrations (FDR-adjusted *p-*value ≤  0.05).**

| Compound | RT  (min) | CAS | Molecular formula | VIP | Ionization mode | Theoretical mass  (*m/z*) | Experimental mass  (*m/z*) | ΔMass (ppm) | Elemental compos (fragment 1) | Theoretical mass  (*m/z*) | Elemental compos (fragment 2) | Theoretical mass  (*m/z*) |
| --- | --- | --- | --- | --- | --- | --- | --- | --- | --- | --- | --- | --- |
| ***Organic acids*** |  |  |  |  |  |  |  |  |  |  |  |  |
| Dihydroorotic acid | 1.1 | 155-54-4 | C_5_H_6_N_2_O_4_ | 1.4105 | [M - H]^¯^ | 157.03220 | 157.03245 | 1.59 | C_4_H_5_N_2_O_2_^¯^ | 112.03455 | C_4_H_5_N_2_O^¯^ | 96.03963 |
| L-Threonic acid | 1.1 | 7306-96-9 | C_4_H_8_O_5_ | 1.1366 | [M - H]^¯^ | 135.03662 | 135.03654 | 0.59 | C_3_HO_3_^¯^ | 83.99312 | C_2_H_3_O^¯^ | 43.01894 |
| 2-Furoate | 1.2 | 88-14-2 | C_5_H_4_O_3_ | 1.0651 | [M - H]^¯^ | 111.01549 | 111.01574 | 2.25 | C_4_H_3_O^¯^ | 66.01784 | CO_2_^¯^ | 43.99038 |
| 2-Oxoglutaric acid | 1.2 | 328-50-7 | C_5_H_6_O_5_ | 1.2423 | [M - H]^¯^ | 145.02097 | 145.02086 | 0.76 | C_4_H_5_O_3_^¯^ | 101.02442 | C_4_HO_2_^¯^ | 80.99820 |
| Citrate | 1.2 | 77-92-9 | C_6_H_8_O_7_ | 1.3559 | [M - H]^¯^ | 191.02645 | 191.02653 | 0.42 | C_6_H_3_O_5_^¯^ | 154.99860 | C_4_HO_3_^¯^ | 96.99312 |
| D-α-Hydroxyglutaric acid | 1.2 | 13095-47-1 | C_5_H_8_O_5_ | 1.1010 | [M - H]^¯^ | 147.03662 | 147.03675 | 0.88 | C_5_HO_3_^¯^ | 108.99312 | C_2_H_3_O^¯^ | 43.01894 |
| Proline | 1.2 | 2517-4-6 | C_5_H_9_NO_2_ | 1.1815 | [M + H]^+^ | 116.07060 | 116.07056 | 0.34 | C_5_H_7_O_2_^+^ | 99.04406 | C_4_H_3_O^+^ | 67.01784 |
| Citraconate | 1.3 | 498-23-7 | C_5_H_6_O_4_ | 1.2795 | [M - H]^¯^ | 129.02606 | 129.02601 | 0.39 | C_5_H_6_O_4_^¯^ | 129.01933 | C_4_H_3_O^¯^ | 67.01894 |
| 2,2-Bis(hydroxymethyl)propionic acid | 1.4 | 4767-03-7 | C_5_H_10_O_4_ | 1.0989 | [M - H]^¯^ | 133.05736 | 133.05743 | 0.53 | C_4_H_6_O_3_^¯^ | 102.03224 | C_4_H_7_O_2_^¯^ | 87.04515 |
| Glucose 1-phosphate | 1.4 | 59-56-3 | C_6_H_13_O_9_P | 1.0352 | [M - H]^¯^ | 259.02916 | 259.02925 | 0.35 | C_5_H_6_O_7_P^¯^ | 208.98566 | C_6_H_3_O_6_^¯^ | 170.99351 |
| Methylmalonate | 1.4 | 516-05-2 | C_4_H_6_O_4_ | 2.4135 | [M - H]^¯^ | 117.02606 | 117.02612 | 0.51 | C_2_H_2_O_2_^¯^ | 58.00606 | CHO_2_^¯^ | 44.99820 |
| 3-Indoleacrylic acid | 2.7 | 29953-71-7 | C_11_H_9_NO_2_ | 1.0306 | [M + H]^+^ | 188.07060 | 188.07045 | 0.80 | C_8_H_9_^+^ | 105.06988 | C_4_H_3_O_2_^+^ | 83.01276 |
| Hippuric acid | 3.4 | 495-69-2 | C_9_H_9_NO_3_ | 1.2365 | [M + H]^+^ | 180.06551 | 180.06553 | 0.11 | C_6_H_9_O^+^ | 97.06479 | C_3_NO^+^ | 65.99744 |
| Taurolithocholic acid sulfate | 4.8 | 15324-65-9 | C_26_H_45_NO_8_S_2_ | 1.1556 | [M + H]^+^ | 564.26593 | 564.26530 | 1.12 | C_26_H_42_NO_5_S^+^ | 480.27782 | C_19_H_32_NO^+^ | 290.24784 |
| Lysyl-valyl-aspartyl-leucine | 6.9 | 140681-91-0 | C_21_H_39_N_5_O_7_ | 1.4768 | [M - H]^¯^ | 472.28440 | 472.28434 | 0.13 | C_5_H_6_N_2_O_3_^¯^ | 142.03839 | C_3_HO_3_^¯^ | 83.99312 |
| Perfluorohexadecanoic acid | 7.8 | 67905-19-5 | C_16_HF_31_O_2_ | 2.2656 | [M + H]^+^ | 814.95543 | 814.95523 | 0.25 | C_6_HF_10_O_2_^+^ | 294.98114 | C_4_HF_4_O^+^ | 140.99580 |
| Adenosine 3'-phosphoric acid | 8.0 | 7532-39-0 | C_29_H_42_N_7_O_17_P_3_S | 1.1206 | [M + H]^+^ | 886.16434 | 886.16443 | 0.10 | C_11_H_22_N_2_O_10_P_2_^+^ | 404.07442 | C_8_H_8_N_5_O^+^ | 190.07234 |
| Palmitic acid | 8.1 | 57-10-3 | C_16_H_32_O_2_ | 1.1759 | [M + H]^+^ | 257.24750 | 257.24759 | 0.35 | C_11_H_22_^+^ | 154.17160 | C_9_H_13_^+^ | 121.10118 |
| (2E,9R)-9-[(3,6-dideoxy-α-L-arabino-hexopyranosyl)oxy]dec-2-enoic acid | 8.2 | 1355681-49-0 | C_16_H_28_O_6_ | 1.6008 | [M - H]^¯^ | 315.18804 | 315.18815 | 0.35 | C_9_H_14_O_3_^¯^ | 170.09484 | C_8_H_11_O_2_^¯^ | 139.07645 |
| 12-Hydroxydodecanoic acid | 8.4 | 505-95-3 | C_12_H_24_O_3_ | 1.6155 | [M - H]^¯^ | 215.17199 | 215.17170 | 1.35 | C_9_H_16_O_2_^¯^ | 156.11558 | C_7_H_7_O_2_^¯^ | 123.04515 |
| Tetranor-12(S)-HETE | 8.7 | 121842-79-3 | C_16_H_26_O_3_ | 1.0652 | [M + H]^+^ | 267.19547 | 267.19565 | 0.67 | C_16_H_21_O^+^ | 229.15869 | C_13_H_15_O^+^ | 187.11174 |
| N-lauroylglycine | 9.2 | 7596-88-5 | C_14_H_27_NO_3_ | 1.2884 | [M - H]^¯^ | 256.19854 | 256.19862 | 0.31 | C_8_H_12_NO_3_^¯^ | 170.08227 | C_2_H_2_O_2_^¯^ | 58.00606 |
| Undec-10-ynoic acid | 9.2 | 2777-65-3 | C_11_H_18_O_2_ | 1.1256 | [M + H]^+^ | 183.13795 | 183.13773 | 1.20 | C_11_H_16_O^+^ | 164.11957 | C_8_H_9_^+^ | 105.06988 |
| Tetranor-12(R)-HETE | 9.3 | 135271-51-1 | C_16_H_26_O_3_ | 1.5662 | [M + H]^+^ | 267.19547 | 267.19532 | 0.56 | C_13_H_15_O^+^ | 187.11174 | C_5_H_8_O_2_^+^ | 100.05188 |
| Tetranor-PGEM | 9.3 | 24769-56-0 | C_16_H_24_O_7_ | 1.5356 | [M + H]^+^ | 329.15947 | 329.15921 | 0.79 | C_9_H_15_O_5_^+^ | 203.09140 | C_6_H_8_O_2_^+^ | 112.05188 |
| Tranexamic acid | 9.3 | 1197-18-8 | C_8_H_15_NO_2_ | 2.0771 | [M + H]^+^ | 158.11755 | 158.11725 | 1.90 | C_5_H_4_O^+^ | 80.02567 | CH_3_O_2_^+^ | 47.01276 |
| Stearolic acid | 9.5 | 506-24-1 | C_18_H_32_O_2_ | 1.2365 | [M + H]^+^ | 281.24750 | 281.24758 | 0.28 | C_10_H_17_O_2_^+^ | 169.12231 | C_6_H_9_O^+^ | 97.06479 |
| 2-Tetrahydrothiopheneacetic acid | 9.8 | 65102-22-9 | C_6_H_10_O_2_S | 1.2561 | [M + H]^+^ | 147.04742 | 147.04754 | 0.82 | C_6_H_8_O_2_^+^ | 112.05188 | C_4_H_3_O_2_^+^ | 83.01276 |
| Oleanolic acid | 10.1 | 508-02-1 | C_30_H_48_O_3_ | 1.5682 | [M + H]^+^ | 457.36762 | 457.36765 | 0.07 | C_19_H_28_O_2_^+^ | 288.20838 | C_9_H_13_O^+^ | 137.09609 |
| Palmitoleic acid | 10.3 | 373-49-9 | C_16_H_30_O_2_ | 1.2698 | [M + H]^+^ | 255.23185 | 255.23167 | 0.71 | C_13_H_24_O_2_^+^ | 212.17708 | C_6_H_13_O_2_^+^ | 117.09101 |
| Phthalic acid | 10.6 | 88-99-3 | C_8_H_6_O_4_ | 1.5892 | [M + H]^+^ | 167.03388 | 167.03393 | 0.30 | C_8_H_3_O_2_^+^ | 131.01276 | C_7_H_4_O^+^ | 104.02567 |
| Stevastelin B | 10.6 | 147334-89-2 | C_34_H_61_N_3_O_9_ | 1.8256 | [M + H]^+^ | 656.44805 | 656.44832 | 0.41 | C_8_H_14_NO^+^ | 140.10699 | C_3_HO_2_^+^ | 68.99711 |
| Tetradecanedioic acid | 10.6 | 821-38-5 | C_14_H_26_O_4_ | 1.2505 | [M + H]^+^ | 259.19038 | 259.19063 | 0.96 | C_12_H_24_O_3_^+^ | 216.17200 | C_8_H_14_O_2_^+^ | 142.09883 |
| Palmitic acid | 10.7 | 57-10-3 | C_16_H_32_O_2_ | 1.4343 | [M - H]^¯^ | 255.23968 | 255.23976 | 0.31 | C_13_H_20_O_2_^¯^ | 208.14688 | C_8_H_12_O_2_^¯^ | 140.08428 |
| Stearic acid | 10.7 | 30399-84-9 | C_18_H_36_O_2_ | 1.0652 | [M + H]^+^ | 285.27880 | 285.27892 | 0.42 | C_11_H_20_O_2_^+^ | 184.14578 | C_5_H_7_O_2_^+^ | 99.04406 |
| Stearate | 11.1 | 30399-84-9 | C_18_H_36_O_2_ | 1.0334 | [M - H]^¯^ | 283.27098 | 283.27086 | 0.42 | C_14_H_24_O_2_^¯^ | 224.17818 | C_13_H_20_O_2_^¯^ | 208.14688 |
| Stearoyllactic acid | 11.1 | 4253-64-9 | C_21_H_40_O_4_ | 1.3567 | [M - H]^¯^ | 355.29211 | 355.29225 | 0.39 | C_10_H_15_O_4_^¯^ | 199.09758 | C_8_H_9_O^¯^ | 121.06589 |
| Selenate | 13.3 | 7783-8-6 | H_2_O_4_Se | 2.5451 | [M - H]^¯^ | 138.91723 | 138.91736 | 0.94 | H_2_O_3_Se^¯^ | 122.92232 | HO_2_Se^¯^ | 106.91958 |
| Methoxyarsonic acid | 13.4 | 70786-76-4 | CH_5_AsO_4_ | 2.6608 | [M - H]^¯^ | 154.93983 | 154.93957 | 1.68 | CH_4_AsO_3_^¯^ | 138.94491 | CH_3_AsO_2_^¯^ | 120.93435 |
| ***Esters*** |  |  |  |  |  |  |  |  |  |  |  |  |
| 2-C-Methyl-D-erythritol 4-phosphate | 1.1 | 206440-72-4 | C_5_H_13_O_7_P | 1.4959 | [M - H]^¯^ | 215.03934 | 215.03959 | 1.16 | C_5_H_6_O_5_P^¯^ | 176.99583 | C_2_H_4_O_4_P^¯^ | 122.98527 |
| Methyl D-glucopyranuronate | 1.1 | 82228-14-6 | C_7_H_12_O_7_ | 1.4727 | [M - H]^¯^ | 207.05775 | 207.05755 | 0.97 | C_7_H_6_O_6_^¯^ | 186.01699 | C_6_H_5_O_4_^¯^ | 141.01933 |
| O-Phosphorylethanolamine | 1.1 | 1071-23-4 | C_2_H_8_NO_4_P | 1.2566 | [M + H]^+^ | 142.02637 | 142.02625 | 0.84 | CH_3_O_4_P^+^ | 109.97635 | CH_2_O_3_P ^+^ | 92.97361 |
| Streptidine 6-phosphate | 1.1 | 73679-08-0 | C_8_H_21_O_7_P | 1.2895 | [M - H]^¯^ | 343.12038 | 343.12058 | 0.58 | C_8_H_15_N_5_O_7_P^¯^ | 324.07146 | C_2_H_4_O_5_P^¯^ | 138.98018 |
| Tybamate | 1.1 | 4268-36-4 | C_13_H_26_N_2_O_4_ | 1.0991 | [M + H]^+^ | 275.19653 | 275.19675 | 0.80 | C_13_H_23_N_2_O_3_^+^ | 255.17032 | C_9_H_15_NO_2_^+^ | 169.10973 |
| N, N-dimethylethanolamine phosphate | 1.2 | 6909-62-2 | C_4_H_12_NO_4_P | 1.0368 | [M - H]^¯^ | 168.04984 | 168.04964 | 1.19 | CH_5_O_4_P^¯^ | 110.99199 | C_4_H_11_NO^¯^ | 88.08351 |
| Pyridostigmine | 3.9 | 155-97-5 | C_9_H_12_N_2_O_2_ | 1.8765 | [M + H]^+^ | 181.09715 | 181.09726 | 0.61 | C_7_H_6_N_2_O^+^ | 134.04746 | C_3_H_6_NO_2_ ^+^ | 88.03930 |
| Methyl 3-Amino-5-(4-Fluoropheyl)Thiophen | 4.8 | 175137-08-3 | C_12_H_10_FNO_2_S | 1.2609 | [M - H]^¯^ | 250.04107 | 250.04115 | 0.32 | C_6_H_5_NO_2_S^¯^ | 155.00355 | C_6_H_4_F^¯^ | 95.02915 |
| Trehalose-6,6-dibehenate | 7.7 | 66758-35-8 | C_56_H_106_O_13_ | 2.7250 | [M + H]^+^ | 987.77061 | 987.77056 | 0.05 | C_34_H_65_O_11_^+^ | 649.45214 | C_24_H_46_O_3_^+^ | 382.34415 |
| Glycerol triricinoleate | 7.8 | 15505-14-3 | C_57_H_104_O_9_ | 1.1765 | [M + H]^+^ | 933.77531 | 933.77549 | 0.19 | C_25_H_45_O_6_^+^ | 441.32107 | C_8_H_13_^+^ | 109.10118 |
| Papulacandin A | 8.2 | 61036-46-2 | C_47_H_66_O_16_ | 1.2568 | [M + H]^+^ | 887.44236 | 887.44216 | 0.23 | C_14_H_21_O_4_^+^ | 253.14344 | C_7_H_7_O_3_^+^ | 139.03897 |
| Octyl isocyanate | 9.5 | 3158-26-7 | C_9_H_17_NO | 1.0083 | [M + H]^+^ | 156.13829 | 156.13846 | 1.09 | C_8_H_13_^+^ | 109.10118 | C_5_H_8_NO^+^ | 98.06004 |
| Pinolenic acid ethyl ester | 9.6 | 493015-74-0 | C_20_H_34_O_2_ | 1.3568 | [M + H]^+^ | 307.26315 | 307.26326 | 0.36 | C_19_H_30_O^+^ | 274.22912 | C_9_H_15_O_2_^+^ | 155.10666 |
| Sedanolide | 10.0 | 6415-59-4 | C_12_H_18_O_2_ | 1.0229 | [M + H]^+^ | 195.13795 | 195.13773 | 1.13 | C_9_H_13_O_2_^+^ | 153.09101 | C_6_H_10_O_2_^+^ | 114.06753 |
| γ-Linolenic acid ethyl ester | 10.2 | 31450-14-3 | C_20_H_34_O_2_ | 1.1071 | [M + H]^+^ | 307.26315 | 307.26347 | 1.04 | C_12_H_20_^+^ | 164.15595 | C_8_H_10_O^+^ | 122.07262 |
| Tridecyl benzenesulfonate | 10.3 | 25474-61-7 | C_19_H_32_O_3_S | 1.2562 | [M - H]^¯^ | 339.20666 | 339.20688 | 0.65 | C_6_H_5_O_3_S^¯^ | 155.99539 | C_13_H_27_O^¯^ | 198.20564 |
| N, N-Diethylaminoethyl methacrylate | 10.5 | 105-16-8 | C_10_H_19_NO_2_ | 1.0365 | [M + H]^+^ | 186.14885 | 186.14876 | 0.48 | C_8_H_14_NO_2_^+^ | 156.10191 | C_4_H_11_NO_2_^+^ | 105.07843 |
| 2,4-Dihydroxyheptadec-16-enyl acetate | 10.7 | 24607-09-8 | C_19_H_36_O_4_ | 1.2508 | [M - H]^¯^ | 327.26081 | 327.26089 | 0.24 | C_17_H_30_O_2_^¯^ | 266.22513 | C_9_H_11_O_4_^¯^ | 183.06628 |
| Trioctyl citrate | 10.8 | 76414-35-2 | C_30_H_56_O_7_ | 1.2137 | [M + H]^+^ | 529.40988 | 529.40968 | 0.38 | C_22_H_38_O_6_ ^+^ | 398.26629 | C_14_H_23_O_4_^+^ | 255.15909 |
| Octadecyl hydrogen sulfate | 10.9 | 143-03-3 | C_18_H_38_O_4_S | 1.5658 | [M - H]^¯^ | 349.24853 | 349.24859 | 0.17 | CH_3_O_4_S^¯^ | 109.97465 | O_3_S^¯^ | 79.95736 |
| Ethyl undecylenate | 11.1 | 692-86-4 | C_13_H_24_O_2_ | 2.4273 | [M + H]^+^ | 213.18490 | 213.18474 | 0.75 | C_11_H_21_O_2_^+^ | 185.15361 | C_6_H_10_O_2_^+^ | 114.06753 |
| Tris(2,3-dibromopropyl) phosphate | 11.1 | 126-72-7 | C_9_H_15_Br_6_O_4_P | 1.2168 | [M + H]^+^ | 692.58809 | 692.58822 | 0.19 | C_8_H_9_Br_4_O_4_P ^+^ | 515.69665 | C_4_H_9_BrO_4_P ^+^ | 230.94163 |
| Distearyl thiodipropionate | 12.3 | 693-36-7 | C_42_H_82_O_4_S | 1.8965 | [M + H]^+^ | 683.60065 | 683.60076 | 0.16 | C_27_H_47_O_3_S ^+^ | 451.32404 | C_11_H_21_O_2_^+^ | 185.15361 |
| ***Amines*** |  |  |  |  |  |  |  |  |  |  |  |  |
| Spermidine | 0.8 | 124-20-9 | C_7_H_19_N_3_ | 1.0523 | [M + H]^+^ | 146.16517 | 146.16526 | 0.62 | C_7_H_12_N^+^ | 110.09643 | C_6_H_8_N^+^ | 94.06513 |
| Glycerophosphoethanolamine | 1.1 | 33049-08-0 | C_5_H_14_NO_6_P | 1.2516 | [M + H]^+^ | 216.06315 | 216.06326 | 0.51 | C_2_H_7_NO_4_P^+^ | 139.98691 | C_7_H_7_NO_2_^+^ | 68.99711 |
| Sn-glycero-3-Phosphoethanolamine | 1.1 | 33049-08-0 | C_5_H_14_NO_6_P | 1.2414 | [M + H]^+^ | 216.06315 | 216.06301 | 0.65 | C_2_H_7_NO_4_P^+^ | 139.98691 | C_2_H_3_O_3_P^+^ | 105.98143 |
| UDP-N-acetyl-D-mannosamine | 1.2 | 26575-17-7 | C_17_H_27_N_3_O_17_P_2_ | 1.1056 | [M + H]^+^ | 608.08884 | 608.08899 | 0.25 | C_8_H_15_NO_8_P^+^ | 284.05298 | C_9_H_9_N_2_O_3_^+^ | 193.06077 |
| Salicylamide | 3.9 | 65-45-2 | C_7_H_7_NO_2_ | 1.3658 | [M + H]^+^ | 138.05495 | 138.05475 | 1.45 | C_7_H_4_NO^+^ | 118.02874 | C_7_H_7_NO_2_^+^ | 68.99711 |
| Patellamide D | 7.9 | 120853-15-8 | C_38_H_48_N_8_O_6_S_2_ | 1.6582 | [M + H]^+^ | 777.32109 | 777.32127 | 0.23 | C_6_H_7_N_2_S^+^ | 139.03245 | C_6_H_12_NO^+^ | 114.09134 |
| Octylamine | 8.0 | 111-86-4 | C_8_H_19_N | 1.0058 | [M + H]^+^ | 130.15902 | 130.15915 | 1.00 | C_7_H_15_^+^ | 99.11683 | C_6_H_8_N^+^ | 94.06513 |
| Stearyldiethanolamine | 9.9 | 10213-78-2 | C_22_H_47_NO_2_ | 1.5562 | [M + H]^+^ | 358.36795 | 358.36786 | 0.25 | C_12_H_22_N^+^ | 180.17468 | C_5_H_12_NO^+^ | 102.09134 |
| N-Ethyl-p-menthane-3-carboxamide | 10.3 | 39711-79-0 | C_13_H_25_NO | 1.2510 | [M + H]^+^ | 212.20089 | 212.20053 | 1.70 | C_12_H_22_N^+^ | 180.17468 | C_9_H_12_O^+^ | 136.08827 |
| Oleoylethanolamide | 10.4 | 111-58-0 | C_20_H_39_NO_2_ | 1.2562 | [M + H]^+^ | 326.30535 | 326.30546 | 0.34 | C_6_H_12_NO^+^ | 114.09134 | C_7_H_15_^+^ | 99.11683 |
| Stearamide | 10.7 | 124-26-5 | C_18_H_37_NO | 2.0562 | [M + H]^+^ | 284.29479 | 284.29452 | 0.95 | C_15_H_31_NO^+^ | 241.24002 | C_7_H_12_N^+^ | 110.09643 |
| Stearoyl ethanolamide | 10.7 | 111-57-9 | C_20_H_41_NO_2_ | 1.1895 | [M + H]^+^ | 328.32100 | 328.32124 | 0.73 | C_19_H_37_N^+^ | 279.29205 | C_10_H_22_NO_2_^+^ | 188.16451 |
| Trilaurylamine | 11.6 | 102-87-4 | C_36_H_75_N | 1.5155 | [M + H]^+^ | 522.59722 | 522.59746 | 0.46 | C_19_H_41_N^+^ | 283.32335 | C_19_H_37_N^+^ | 279.29205 |
| ***Amino acids*** |  |  |  |  |  |  |  |  |  |  |  |  |
| 2-Methylserine | 1.0 | 5424-29-3 | C_4_H_9_NO_3_ | 1.3738 | [M - H]^¯^ | 118.05769 | 118.05754 | 1.27 | C_4_H_5_O_3_^¯^ | 101.02442 | C_3_H_6_NO_2_^¯^ | 88.04040 |
| Symmetric dimethylarginine | 1.0 | 30344-00-4 | C_8_H_18_N_4_O_2_ | 1.0456 | [M + H]^+^ | 203.15025 | 203.15054 | 1.43 | C_8_H_17_N_4_^+^ | 169.14477 | C_3_H_9_N_2_O_2_^+^ | 105.06585 |
| Val-lys | 1.0 | 22677-62-9 | C_11_H_23_N_3_O_3_ | 2.0521 | [M + H]^+^ | 246.18121 | 246.18146 | 1.02 | C_11_H_19_N_2_O_2_^+^ | 211.14410 | C_6_H_15_N_2_O_2_^+^ | 147.11280 |
| Hypotaurine | 1.1 | 300-84-5 | C_2_H_7_NO_2_S | 1.0535 | [M + H]^+^ | 110.02702 | 110.02713 | 1.00 | C_2_H_3_NO_2_S^+^ | 104.98790 | C_2_H_3_NOS^+^ | 88.99299 |
| Taurine | 1.1 | 107-35-7 | C_2_H_7_NO_3_S | 1.1035 | [M + H]^+^ | 126.02194 | 126.02189 | 0.40 | C_2_H_5_O_3_S^+^ | 108.99539 | C_2_H_4_NOS^+^ | 90.00081 |
| 4-Oxoproline | 1.2 | 2002-02-0 | C_5_H_7_NO_3_ | 1.5236 | [M - H]^¯^ | 119.06551 | 119.06545 | 0.50 | C_4_H_6_NO^¯^ | 84.04439 | CO_2_^¯^ | 43.99038 |
| Tripalmitoyl-S-glyceryl-cysteinyl-seryl-serine | 7.4 | 98633-82-0 | C_60_H_113_N_3_O_11_S | 1.4833 | [M + H]^+^ | 1084.81685 | 1084.81699 | 0.13 | C_25_H_48_N_3_O_6_S^+^ | 518.32583 | C_8_H_13_N_3_O_6_^+^ | 247.07989 |
| Leu-val-val-tyr-pro-trp-thr | 7.9 | 137201-62-8 | C_45_H_64_N_8_O_10_ | 1.2523 | [M + H]^+^ | 877.48181 | 877.48176 | 0.06 | C_31_H_34_N_6_O_8_^+^ | 618.24326 | C_10_H_22_N_3_O^+^ | 200.17574 |
| N-Palmitoylglycin | 11.4 | 2441-41-0 | C_18_H_35_NO_3_ | 1.2552 | [M + H]^+^ | 314.26897 | 314.26886 | 0.35 | C_18_H_30_NO_2_^+^ | 292.22711 | C_8_H_16_NO_3_^+^ | 174.11247 |
| Ser-ile-lys-val-ala-val | 11.6 | 655230-51-6 | C_28_H_53_N_7_O_8_ | 1.0559 | [M + H]^+^ | 616.40283 | 616.40261 | 0.36 | C_10_H_22_N_3_O^+^ | 200.17574 | C_7_H_17_N_2_O_2_^+^ | 161.12845 |
| Prolylleucine | 13.3 | 52899-07-7 | C_11_H_20_N_2_O_3_ | 1.6825 | [M + H]^+^ | 229.15466 | 229.15442 | 1.05 | C_9_H_16_N_2_O_3_^+^ | 200.11554 | C_7_H_11_N_2_O^+^ | 139.08659 |
| ***Carbohydrates*** |  |  |  |  |  |  |  |  |  |  |  |  |
| D-Galactose | 1.0 | 3646-73-9 | C_6_H_12_O_6_ | 1.3969 | [M - H]^¯^ | 179.06283 | 179.06245 | 2.12 | C_6_HO_4_^¯^ | 136.98803 | C_5_H_4_O_3_^¯^ | 112.01659 |
| Lactose | 1.0 | 14641-93-1 | C_12_H_22_O_11_ | 1.5451 | [M + H]^+^ | 343.12348 | 343.12352 | 0.13 | C_4_H_8_O_3_^+^ | 104.04680 | C_3_H_4_O_3_^+^ | 88.01550 |
| Maltose | 1.0 | 69-79-4 | C_12_H_22_O_11_ | 1.5769 | [M + H]^+^ | 343.12348 | 343.12324 | 0.70 | C_7_H_11_N_2_O_6_^+^ | 219.06116 | C_5_H_11_N_2_O_4_^+^ | 163.07133 |
| D-Erythrose | 1.1 | 583-50-6 | C_4_H_8_O_4_ | 1.1428 | [M - H]^¯^ | 119.04171 | 119.04167 | 0.34 | C_4_H_4_O_3_^+^ | 100.01659 | C_4_H_3_O_2_^+^ | 83.01385 |
| D-Glucose | 1.1 | 492-62-6 | C_6_H_12_O_6_ | 1.0061 | [M + H]^+^ | 181.07066 | 181.07058 | 0.44 | C_6_H_7_O_4_^+^ | 143.03389 | C_4_H_8_O_3_^+^ | 104.04680 |
| D-Xylose | 1.1 | 6763-34-4 | C_5_H_10_O_5_ | 1.2482 | [M - H]^¯^ | 149.05227 | 149.05204 | 1.54 | C_5_H_4_O_3_^¯^ | 112.01659 | C_4_H_4_O_3_^+^ | 100.01659 |
| Raffinose | 1.1 | 512-69-6 | C_18_H_32_O_16_ | 1.5448 | [M + H]^+^ | 505.17631 | 505.17674 | 0.85 | C_4_H_7_O_4_^+^ | 119.03389 | C_4_H_9_O_3_^+^ | 105.05462 |
| UDP-Galactose | 1.3 | 2956-16-3 | C_15_H_24_N_2_O_17_P_2_ | 1.3655 | [M - H]^¯^ | 565.05447 | 565.05434 | 0.23 | C_6_H_9_O_5_^¯^ | 161.04555 | C_5_H_9_O_4_^¯^ | 149.04555 |
| ***Alcohols*** |  |  |  |  |  |  |  |  |  |  |  |  |
| D-Mannitol | 1.0 | 69-65-8 | C_6_H_14_O_6_ | 1.2007 | [M - H]^¯^ | 181.07848 | 181.07856 | 0.44 | C_6_H_8_O_4_^¯^ | 144.04281 | C_4_H_9_O_3_^¯^ | 105.05572 |
| Maltitol | 1.0 | 585-88-6 | C_12_H_24_O_11_ | 1.3513 | [M - H]^¯^ | 183.18216 | 183.18208 | 0.44 | C_6_H_6_O_6_^¯^ | 174.01699 | C_6_H_8_O_4_^¯^ | 144.04281 |
| Octylthiol | 1.1 | 111-88-6 | C_8_H_18_S | 1.5899 | [M + H]^+^ | 147.12019 | 147.12025 | 0.41 | C_7_H_13_S^+^ | 129.07325 | C_5_H_9_S^+^ | 101.04195 |
| Sphinganine | 8.9 | 764-22-7 | C_18_H_39_NO_2_ | 1.5562 | [M + H]^+^ | 302.30535 | 302.30563 | 0.93 | C_7_H_12_NO^+^ | 126.09134 | C_6_H_11_O_2_^+^ | 115.07536 |
| Triethylene glycol | 10.0 | 112-27-6 | C_6_H_14_O_4_ | 1.2317 | [M + H]^+^ | 151.09648 | 151.09665 | 1.13 | C_6_H_11_O_2_^+^ | 115.07536 | C_4_H_10_O_2_^+^ | 90.06753 |
| Tridecaethylene glycol | 10.1 | 17598-96-8 | C_28_H_58_O_15_ | 1.0236 | [M + H]^+^ | 635.38484 | 635.38467 | 0.27 | C_6_H_13_O_4_^+^ | 149.08084 | C_5_H_10_O_3_^+^ | 118.06245 |
| Palmitoyl ethanolamide | 10.2 | 544-31-0 | C_18_H_37_NO_2_ | 1.2689 | [M + H]^+^ | 300.28970 | 300.28989 | 0.63 | C_7_H_16_NO_2_^+^ | 146.11756 | C_7_H_10_N^+^ | 108.08078 |
| Quinuclidinol | 10.3 | 1619-34-7 | C_7_H_13_NO | 1.1209 | [M + H]^+^ | 128.10699 | 128.10657 | 3.28 | C_7_H_12_NO^+^ | 126.09134 | C_6_H_11_NO^+^ | 113.08352 |
| ***Glycosides*** |  |  |  |  |  |  |  |  |  |  |  |  |
| Methyl-2-O-Methylfucoside | 1.2 | 59981-27-0 | C_8_H_16_O_5_ | 1.2256 | [M - H]^¯^ | 191.09922 | 191.09936 | 0.73 | C_6_H_8_O_4_^¯^ | 144.04281 | C_6_H_11_O_2_^¯^ | 115.07645 |
| Rebaudioside M | 7.6 | 1220616-44-3 | C_56_H_90_O_33_ | 1.1769 | [M + H]^+^ | 1291.54371 | 1291.54353 | 0.14 | C_7_H_13_O_6_^+^ | 193.07066 | C_6_H_9_O_5_^+^ | 161.04445 |
| Ophiopogonin D | 7.7 | 41753-55-3 | C_44_H_70_O_16_ | 1.2256 | [M + H]^+^ | 855.47366 | 855.47357 | 0.11 | C_7_H_12_O_6_^+^ | 192.06284 | C_5_H_12_O_4_^+^ | 136.07301 |
| Ziziphin | 8.2 | 73667-51-3 | C_51_H_80_O_18_ | 1.5865 | [M + H]^+^ | 981.54174 | 981.54154 | 0.20 | C_11_H_15_O_9_^+^ | 291.07106 | C_8_H_15_O_6_^+^ | 207.08631 |
| Psychosine | 11.2 | 2238-90-6 | C_24_H_47_NO_7_ | 1.5825 | [M - H]^¯^ | 460.33470 | 460.33451 | 0.41 | C_10_H_18_NO_2_^¯^ | 184.13430 | C_6_H_8_O_4_^¯^ | 144.04281 |
| ***Acylamides*** |  |  |  |  |  |  |  |  |  |  |  |  |
| Oleamide | 10.3 | 301-02-0 | C_18_H_35_NO | 1.0036 | [M + H]^+^ | 282.27914 | 282.27906 | 0.28 | C_17_H_33_O^+^ | 253.25259 | C_15_H_27_^+^ | 207.21073 |
| Octadecylacrylamide | 10.9 | 1506-54-3 | C_21_H_41_NO | 1.0020 | [M + H]^+^ | 324.32609 | 324.32650 | 1.26 | C_15_H_27_^+^ | 207.21073 | C_9_H_20_N^+^ | 142.15903 |
| Oleyl anilide | 10.9 | 5429-85-6 | C_24_H_39_NO | 1.1924 | [M + H]^+^ | 358.31044 | 358.31002 | 1.17 | C_14_H_27_^+^ | 195.21073 | C_7_H_8_NO^+^ | 122.06004 |
| Oleoyl ethylamide | 11.1 | 85075-82-7 | C_20_H_39_NO | 1.5562 | [M + H]^+^ | 310.31044 | 310.31011 | 1.06 | C_19_H_37_NO^+^ | 295.28697 | C_17_H_33_O^+^ | 253.25259 |
| ***Ketones*** |  |  |  |  |  |  |  |  |  |  |  |  |
| Robinetin | 0.9 | 490-31-3 | C_15_H_10_O_7_ | 2.2419 | [M + H]^+^ | 303.04992 | 303.04986 | 0.20 | C_11_H_6_O_3_^+^ | 186.03115 | C_6_H_4_O_3_^+^ | 124.01550 |
| α-Phthalimidopropiophenone | 1.0 | 19437-20-8 | C_17_H_13_NO_3_ | 1.5477 | [M + H]^+^ | 280.09681 | 280.09662 | 0.68 | C_16_H_11_NO_2_^+^ | 249.07843 | C_11_H_8_NO_2_^+^ | 186.05495 |
| Persin | 10.2 | 60640-59-7 | C_23_H_40_O_4_ | 2.6392 | [M + H]^+^ | 381.29993 | 381.29970 | 0.60 | C_11_H_19_O_3_^+^ | 199.13287 | C_7_H_10_O_4_^+^ | 158.05736 |
| Oxidized latia luciferin | 10.3 | 17283-81-7 | C_13_H_22_O | 1.1959 | [M + H]^+^ | 195.17434 | 195.17445 | 0.56 | C_11_H_17_O^+^ | 165.12739 | C_7_H_10_O^+^ | 110.07262 |
| ***Alkanes*** |  |  |  |  |  |  |  |  |  |  |  |  |
| Terbufos-oxon-sulfoxide | 1.3 | 56165-57-2 | C_9_H_21_O_4_PS_2_ | 2.0439 | [M + H]^+^ | 289.06916 | 289.06933 | 0.59 | C_5_H_14_O_4_PS_2_^+^ | 233.00656 | C_4_H_10_O_3_P^+^ | 137.03621 |
| 1,1,3,3-Tetraethoxypropane | 10.5 | 122-31-6 | C_11_H_24_O_4_ | 1.2669 | [M + H]^+^ | 221.17473 | 221.17423 | 2.26 | C_11_H_19_O_3_^+^ | 199.13287 | C_8_H_17_O_3_^+^ | 161.11722 |
| Octylbenzene | 11.1 | 2189-60-8 | C_14_H_22_ | 1.6565 | [M + H]^+^ | 191.17942 | 191.17937 | 0.26 | C_9_H_7_^+^ | 115.05423 | C_8_H_6_^+^ | 102.04640 |
| ***Purines*** |  |  |  |  |  |  |  |  |  |  |  |  |
| 7-Methylxanthine | 1.1 | 552-62-5 | C_6_H_6_N_4_O_2_ | 1.3071 | [M - H]^¯^ | 165.04852 | 165.04847 | 0.30 | C_4_H_3_N_2_O_2_^¯^ | 111.01890 | C_3_H_5_N_2_^¯^ | 69.04472 |
| Hypoxanthine | 1.2 | 68-94-0 | C_5_H_4_N_4_O | 1.6658 | [M + H]^+^ | 137.04578 | 137.04592 | 1.02 | C_4_N_3_O^+^ | 106.00359 | C_3_HN_3_^+^ | 79.01650 |
| ***Nucleosides*** |  |  |  |  |  |  |  |  |  |  |  |  |
| 2'-Deoxyinosine | 1.2 | 890-38-0 | C_10_H_12_N_4_O_4_ | 1.4556 | [M + H]^+^ | 253.09313 | 253.09339 | 1.03 | C_5_H_4_N_3_O^+^ | 122.03489 | C_4_H_6_O_3_^+^ | 102.03115 |
| N-(Methylamino)cytidine | 3.3 | 100997-69-1 | C_10_H_16_N_4_O_5_ | 1.0591 | [M - H]^¯^ | 271.11152 | 271.11145 | 0.26 | C_4_H_7_O_3_^¯^ | 103.04007 | C_3_H_5_O_2_^¯^ | 73.02840 |
| ***Phenols*** |  |  |  |  |  |  |  |  |  |  |  |  |
| Phloroglucinol | 3.9 | 108-73-6 | C_6_H_6_O_3_ | 1.0036 | [M + H]^+^ | 127.03897 | 127.03875 | 1.73 | C_6_H_4_O_2_^+^ | 108.02058 | C_6_H_4_^+^ | 76.03075 |
| Apomine | 10.0 | 126411-13-0 | C_28_H_52_O_7_P_2_ | 2.4252 | [M + H]^+^ | 563.32610 | 563.32632 | 0.39 | C_3_H_10_O_3_P^+^ | 125.03621 | C_3_H_7_O_2_P^+^ | 106.01782 |
| ***Triterpenoid saponins*** |  |  |  |  |  |  |  |  |  |  |  |  |
| Hederacolchiside A | 7.7 | 68027-15-6 | C_47_H_76_O_17_ | 1.3662 | [M + H]^+^ | 913.51552 | 913.51528 | 0.26 | C_15_H_23_O_11_^+^ | 379.12349 | C_5_H_12_O_4_^+^ | 136.07301 |
| ***Pyrazoles*** |  |  |  |  |  |  |  |  |  |  |  |  |
| 4-Chloropyrazole | 0.8 | 15878-00-9 | C_3_H_3_ClN_2_ | 1.0822 | [M + H]^+^ | 89.00267 | 89.00249 | 2.02 | C_3_H_3_N_2_^+^ | 67.02907 | CH_3_N_2_^+^ | 43.02907 |
| ***Porphinoids*** |  |  |  |  |  |  |  |  |  |  |  |  |
| Sirohaem | 7.7 | 52553-42-1 | C_42_H_44_FeN_4_O_16_ | 2.6392 | [M + H]^+^ | 915.22212 | 915.22224 | 0.13 | C_21_H_23_N_2_O_6_^+^ | 399.15506 | C_12_H_16_NO_4_^+^ | 238.10738 |
| ***Other compounds*** |  |  |  |  |  |  |  |  |  |  |  |  |
| Phosphonoacetaldehyde | 1.1 | 16051-76-6 | C_2_H_5_O_4_P | 1.1041 | [M + H]^+^ | 124.99982 | 124.99976 | 0.48 | C_2_H_4_O_3_P^+^ | 106.98926 | C_2_H_2_O_2_P^+^ | 88.97869 |
| Decylpentaglycol | 1.2 | 23244-49-7 | C_20_H_42_O_6_ | 1.0275 | [M - H]^¯^ | 378.29759 | 378.29746 | 0.34 | C_12_H_24_O_6_^+^ | 264.15674 | C_10_H_19_O^+^ | 155.14304 |
| Propionylcarnitine | 1.2 | 17298-37-2 | C_10_H_19_NO_4_ | 2.8875 | [M + H]^+^ | 218.13868 | 218.13852 | 0.73 | C_7_H_15_NO_3_^+^ | 161.10464 | C_4_H_10_NO_2_^+^ | 104.07060 |
| Tetramesitylporphyrin | 7.9 | 56396-12-4 | C_56_H_54_N_4_ | 1.2336 | [M + H]^+^ | 783.44212 | 783.44224 | 0.15 | C_27_H_28_N^+^ | 366.22163 | C_16_H_15_N_2_^+^ | 235.12297 |
| Undecylbenzene | 10.3 | 67774-74-7 | C_17_H_28_ | 1.2177 | [M + H]^+^ | 233.22637 | 233.22655 | 0.77 | C_16_H_25_^+^ | 217.19508 | C_10_H_12_^+^ | 132.09335 |
| Cetyl sulfate | 10.5 | 143-02-2 | C_16_H_34_O_4_S | 1.6692 | [M - H]^¯^ | 321.21723 | 321.21745 | 0.68 | C_16_H_33_O_3_S^+^ | 305.21449 | C_12_H_19_^+^ | 163.14813 |
| Ubiquinol | 11.1 | 992-78-9 | C_49_H_78_O_4_ | 1.0061 | [M + H]^+^ | 731.59728 | 731.59753 | 0.34 | C_23_H_34_O_4_^+^ | 374.24516 | C_9_H_13_O_4_^+^ | 185.08084 |
| Platelet-activating factor | 12.6 | 65154-06-5 | C_26_H_54_NO_7_P | 2.0036 | [M + H]^+^ | 524.37106 | 524.37172 | 1.26 | C_19_H_40_O_5_P^+^ | 379.26079 | C_4_H_11_NO_3_P^+^ | 152.04711 |

$$\text{ΔMass (ppm)=│(Experimental mass }\text{（}\text{m/z}\text{）}\text{-Theoretical mass}\text{（}\text{m/z}\text{）}\text{)/(Theoretical mass}\text{（}\text{m/z}\text{）}\text{)│}$$

**Table S3 Summary of the method validation performance characteristics as determined for the untargeted metabolomics method.**

| Compound | Family | Linear range  (μg L^-1^) | Correlation coefficients (R^2^) | Overall  recovery % ^a^ | LOD ^b^ (μg L ^-1^) | LOQ ^b^ (μg L ^-1^) | Precisions (RSD%, n = 6) | | |
| --- | --- | --- | --- | --- | --- | --- | --- | --- | --- |
|  |  |  |  |  |  |  | Intra-day | Inter-day | |
| D-Galactose | Carbohydrates | 100-1000 | 0.9986 | 97.48 | 34.65 | 98.89 | 1.46 | | 0.99 |
| D-Glucose | Carbohydrates | 100-1000 | 0.9989 | 101.78 | 29.38 | 80.67 | 2.42 | | 3.24 |
| Lactose | Carbohydrates | 25-750 | 0.9999 | 98.87 | 6.89 | 15.75 | 2.31 | | 3.76 |
| Maltose | Carbohydrates | 100-1000 | 0.9982 | 97.92 | 22.36 | 58.75 | 2.93 | | 3.53 |
| Raffinose | Carbohydrates | 25-750 | 0.9994 | 93.40 | 9.34 | 23.42 | 6.20 | | 4.36 |
| UDP-Galactose | Carbohydrates | 100-1000 | 0.9996 | 96.45 | 16.78 | 48.45 | 1.25 | | 2.63 |
| Ethyl undecylenate | Esters | 5-300 | 0.9992 | 105.82 | 2.12 | 7.79 | 3.92 | | 4.60 |
| Glycerol triricinoleate | Esters | 5-300 | 0.9999 | 93.50 | 1.98 | 5.64 | 3.25 | | 4.57 |
| Octyl isocyanate | Esters | 10-500 | 0.9979 | 95.36 | 3.42 | 9.18 | 5.92 | | 6.32 |
| Trioctyl citrate | Esters | 10-500 | 0.9996 | 94.74 | 2.88 | 8.27 | 2.62 | | 6.11 |
| γ-Linolenic acid ethyl ester | Esters | 10-500 | 0.9998 | 94.10 | 3.56 | 8.02 | 3.49 | | 4.26 |
| Adenosine 3'-phosphoric acid | Organic acids | 5-300 | 0.9994 | 90.22 | 1.92 | 6.36 | 2.41 | | 3.93 |
| Glucose 1-phosphate | Organic acids | 10-500 | 0.9989 | 108.91 | 2.23 | 5.08 | 0.77 | | 1.82 |
| Hippuric acid | Organic acids | 5-300 | 0.9988 | 92.12 | 2.86 | 8.35 | 4.97 | | 3.86 |
| Palmitic acid | Organic acids | 5-300 | 0.9990 | 92.31 | 1.87 | 5.40 | 1.68 | | 2.68 |
| Stearic acid | Organic acids | 10-500 | 0.9989 | 95.36 | 2.02 | 5.86 | 5.30 | | 6.23 |
| Octylamine | Amines | 5-300 | 0.9997 | 95.83 | 1.08 | 2.56 | 1.82 | | 2.24 |
| Oleoylethanolamide | Amines | 5-300 | 0.9996 | 95.69 | 0.98 | 2.39 | 1.23 | | 2.03 |
| Spermidine | Amines | 5-300 | 0.9995 | 92.36 | 1.32 | 3.84 | 2.40 | | 4.65 |
| Hypotaurine | Amino acids | 10-500 | 0.9996 | 97.86 | 3.07 | 7.65 | 1.70 | | 3.01 |
| Taurine | Amino acids | 10-500 | 0.9993 | 96.62 | 3.89 | 9.75 | 1.17 | | 2.83 |
| Octadecylacrylamide | Acylamides | 10-500 | 0.9986 | 94.36 | 4.21 | 9.46 | 2.70 | | 2.98 |
| Oleamide | Acylamides | 10-500 | 0.9999 | 93.52 | 3.15 | 8.06 | 2.68 | | 3.25 |
| Ziziphin | Glycosides | 10-500 | 0.9997 | 98.32 | 2.98 | 8.69 | 2.16 | | 3.28 |
| Robinetin | Ketones | 5-300 | 0.9994 | 92.98 | 1.22 | 3.04 | 5.62 | | 6.02 |
| Hypoxanthine | Purines | 25-750 | 0.9993 | 96.56 | 7.62 | 20.36 | 0.92 | | 1.51 |

*^a^* Average of three concentration levels-LOQ, 2 × LOQ, 4 × LOQ.

*^b^* LOQ, limit of quantification (S/N = 10); LOD, limit of detection (S/N = 3).

**Table S4 Comparison of the method performance (limits of detection and quantification) and determination of metabolites in the present work and the literatures.**

| Sample | Measurement method | Analysis software and method | Quantification | Number  of metabolites | LOD ^a^  (μg L^-1^) | LOQ ^a^  (μg L^-1^) | Ref. |
| --- | --- | --- | --- | --- | --- | --- | --- |
| Goat milk yoghurt | GC-MS ^b^ | XCMS | No quantification | 129 | - | - | [6] |
| Cow and goat milk  yoghurt | GC-MS ^b^ | XCMS | No quantification | 105 | - | - | [16] |
| Yoghurts from sheep's  and goats' milk | GC-MS ^b^ | Co-chromatography approach  with analytical standards | No quantification | 92 | - | - | [24] |
| Milk fermented by  *Lactobacillus plantarum* P9 | UPLC-Q-TOF-MS/MS ^c^ | MS^E^ method  Progenesis QI | No quantification | 35 | - | - | [25] |
| Brown fermented milk  and brown milk | UPLC-QTOF MS^E d^ | MS^E^ method  MassLynx 4.1  Progenesis QI | No quantification | 43 | - | - | [26] |
| Fermented milk | UPLC-MS/MS ^e^ | Compound Discoverer 3.0 | No quantification | 179 | - | - | [27] |
| Fermented goat milk | UHPLC-Q-Orbitrap HRMS ^f^ | Metabolites annotated  signpost (Fig. 2) | External standard | 142 | 0.98-34.65 | 2.39-98.98 | Present  work |

^a^ LOQ, limit of quantification (S/N=10); LOD, limit of detection (S/N = 3).

^b^ GC-MS stands for gas chromatography-mass spectrometry.

^c^ UPLC-Q-TOF-MS/MS stands for ultra-performance liquid chromatography coupled with time-of-flight mass spectrometry.

^d^ UPLC-QTOF MS^E^ stands for ultra-performance liquid chromatography coupled with a quadrupole-time-of-flight mass spectrometry^Elevated Energy^.

^e^ UPLC-MS/MS stands for ultra-high-performance liquid chromatography coupled with tandem mass spectrometry.

^f^ UHPLC-Q-Orbitrap HRMS stands for ultrahigh-performance liquid chromatography coupled to hybrid quadrupole-Orbitrap high-resolution mass spectrometry.

**Table S5 High-content differentially expressed proteins (FDR-adjusted *p*-value ≤  0.05) of fermented goat milk with two final benzoic acid concentrations (0.00 mg L^-1^ and 40.00 mg L^-1^).**

| Protein IDs | Protein names | Gene names | Length | Mass (Da) | VIP scores | Fold-change | |
| --- | --- | --- | --- | --- | --- | --- | --- |
|  |  |  |  |  |  | 40 ppm / 0 ppm | *p*-value |
| A0A0P0EVY5 | α-S1-casein | CSN1S1 | 212 | 24,002 | 6.5911 | 2.2754 | 0.0148 |
| C6K2K5 | Lactoferrin | Lf | 711 | 77,547 | 5.3557 | 2.4007 | 0.0427 |
| A0A452DLQ6 | WWC family member 3 | WWC3 | 1,087 | 122,520 | 5.3557 | 3.4501 | 0.0266 |
| A0A452DMF8 | Keratin 5 | KRT5 | 561 | 60,238 | 5.3237 | 4.1668 | 0.0190 |
| A0A452DP20 | Zinc finger protein 8 | ZNF8 | 558 | 61,227 | 4.3755 | 2.4164 | 0.0318 |
| A0A452DY37 | β-2-microglobulin | LOC102168547 | 124 | 14,006 | 4.2802 | 2.6170 | 0.0284 |
| A0A452DYG7 | Short palate, lung and nasal epithelium carcinoma-associated protein 2B | LOC102171351 | 161 | 17,361 | 4.2784 | 3.4065 | 0.0378 |
| A0A452E1I0 | ADAM metallopeptidase with thrombospondin type 1 motif 7 | ADAMTS7 | 1,647 | 178,982 | 4.2784 | 0.3156 | 0.0010 |
| A0A452E2F6 | α-1-acid glycoprotein | AGP | 202 | 23,131 | 4.2770 | 2.2693 | 0.0235 |
| A0A452E2Y0 | Clusterin | LOC102182127 | 439 | 50,893 | 3.9092 | 3.7912 | 0.0007 |
| A0A452E9Y6 | Lactoperoxidase | LPO | 712 | 80,366 | 3.8016 | 2.4489 | 0.0035 |
| A0A452ECD3 | Serum amyloid A protein | LOC100860781 | 128 | 14,278 | 3.7945 | 5.3553 | 0.0366 |
| A0A452EDG7 | Polymeric immunoglobulin receptor | PIGR | 758 | 82,602 | 3.7912 | 4.3267 | 0.0155 |
| A0A452EGX6 | Glycosylation-dependent cell adhesion molecule 1 | GLYCAM1 | 152 | 16,762 | 3.6833 | 2.3330 | 0.0037 |
| A0A452EIE6 | Perilipin | PLIN2 | 378 | 41,306 | 3.6827 | 3.4830 | 0.0137 |
| A0A452EQX6 | Diazepam binding inhibitor, acyl-CoA binding protein | DBI | 87 | 10,040 | 3.6335 | 3.2900 | 0.0395 |
| A0A452ES93 | Syndecan | SDC2 | 197 | 21,913 | 3.5380 | 2.1019 | 0.0189 |
| A0A452EZ30 | Leucine rich α-2-glycoprotein 1 | LRG1 | 353 | 39,058 | 3.5002 | 4.2364 | 0.0004 |
| A0A452F449 | Mammaglobin-A | LOC102180465 | 94 | 10,659 | 3.4528 | 0.2036 | 0.0116 |
| A0A452FGF3 | PCF11 cleavage and polyadenylation factor subunit | PCF11 | 1,512 | 167,938 | 3.4511 | 4.2883 | 0.0302 |
| A0A452FH78 | α-2-HS-glycoprotein | AHSG | 359 | 38,185 | 3.4505 | 2.3754 | 0.0001 |
| A0A452FI14 | Apolipoprotein A1 | APOA1 | 265 | 30,322 | 3.4501 | 3.3588 | 0.0361 |
| A0A452FQC0 | Cathelicidin-1 | LOC102169231 | 155 | 17,606 | 3.2918 | 0.1913 | 0.0002 |
| A0A452FSL4 | Family with sequence similarity 217 member A | FAM217A | 472 | 52,736 | 2.6633 | 2.1646 | 0.0369 |
| A0A452G7G8 | Joining chain of multimeric IgA and IgM | JCHAIN | 158 | 18,034 | 2.6070 | 2.1576 | 0.0239 |
| A0A6H0DV96 | α-S2-casein | CSN1S2 | 223 | 26,361 | 2.4491 | 3.1544 | 0.0353 |
| A0A7S6C9X0 | β-casein | CSN2 | 222 | 24,893 | 2.3747 | 3.1279 | 0.0005 |
| A3EY52 | Butyrophilin subfamily 1 member A1 | BTN1A1 | 526 | 59,266 | 2.3569 | 2.1046 | 0.0229 |
| A5JSS8 | α-lactalbumin (Lactose synthase B protein) | LALBA | 142 | 16,255 | 2.2869 | 2.1605 | 0.0344 |
| A9YUB7 | Osteopontin | OPN | 277 | 30,883 | 2.2362 | 0.1282 | 0.0012 |
| B2YKX6 | α-lactalbumin (Fragment) | ALA | 122 | 13,858 | 2.1886 | 3.1484 | 0.0164 |
| B2YKY4 | β-lactoglobulin (Fragment) | BLG | 24 | 2,674 | 2.1883 | 2.8348 | 0.0076 |
| B3VHM9 | Albumin | ALB | 90 | 10,055 | 2.1649 | 2.1177 | 0.0248 |
| D6PX62 | Cysteine-rich secretory protein 3 | CRISP3 | 244 | 27,326 | 2.1595 | 3.1210 | 0.0138 |
| A0A452EIH8 | Nucleobindin 1 | NUCB1 | 471 | 54,653 | 2.1586 | 4.1847 | 0.0012 |
| P02670 | Kappa-casein (Kappa-CN) | CSN10 | 192 | 21,441 | 2.1576 | 2.1345 | 0.0001 |
| P02756 | β-lactoglobulin (β-LG) | LGB | 180 | 19,976 | 2.1540 | 5.0835 | 0.0179 |
| P82018 | Cathelicidin-2 (Bactenecin-5) | BAC5 | 176 | 19,846 | 2.1477 | 4.0778 | 0.0110 |
| Q6UZ47 | Antiluteolysin | LOC100861164 | 195 | 22,354 | 2.1283 | 0.1056 | 0.0033 |
| Q7YRX4 | Kappa-casein (Fragment) | CSN3 | 162 | 18,093 | 2.1282 | 2.4366 | 0.0008 |
| Q9XSQ8 | MAP28 protein | MAP28 | 158 | 17,745 | 1.9075 | 3.0757 | 0.0024 |
| A0A452EK88 | β-galactosidase | GLB1 | 646 | 72,642 | 1.8404 | 2.1271 | 0.0167 |
| A0A452F1U4 | Glutamate decarboxylase 1 | GAD1 | 594 | 66,827 | 1.8348 | 3.0648 | 0.0195 |
| E9NRZ3 | β-1,4-galactosyltransferase 1 | B4GALT1 | 402 | 44,758 | 1.8348 | 0.3815 | 0.0137 |
| A0A452G4K3 | ATP-dependent 6-phosphofructokinase | PFKM | 780 | 85,595 | 1.7507 | 4.0851 | 0.0065 |
| CTHL2 | Cathelicidin-2 | CATHL2 | 176 | 19,846 | 1.6169 | 1.3820 | 0.0261 |
| A0A452E555 | MAP34-B protein | MAP34-B | 165 | 18,770 | 1.6154 | 1.2769 | 0.0431 |
| A0A452EN89 | Secreted phosphoprotein 1 | SPP1 | 279 | 31,115 | 1.6147 | 0.0965 | 0.0123 |
| A0A452EU55 | 2-phospho-D-glycerate hydro-lyase | ENO1 | 434 | 47,312 | 1.6147 | 1.1314 | 0.0000 |
| A0A452DYR7 | Cystatin-C | CST3 | 164 | 18,025 | 1.4833 | 0.3470 | 0.0074 |
| Q6S4N9 | Fatty acid binding protein 3 | H-FABP | 133 | 14,762 | 1.4180 | 0.2930 | 0.0012 |
| A0A452FJ15 | Nucleoside diphosphate kinase | NME2 | 152 | 17,265 | 1.4166 | 1.4767 | 0.0251 |
| A0A452FYQ9 | 60S ribosomal protein L40 | UBA52 | 128 | 14,728 | 1.4164 | 0.0908 | 0.0074 |
| K9LQQ8 | Adipocyte-type fatty acid-binding protein | FABP3 | 132 | 14,689 | 1.3562 | 0.1802 | 0.0045 |
| A2SY11 | Lipoprotein lipase | LPL | 478 | 53,415 | 1.3551 | 0.1700 | 0.0120 |
| A0A452FKN8 | Transketolase | TKT | 623 | 67,891 | 1.3335 | 1.1700 | 0.0002 |
| A0A452EU77 | Pyruvate kinase | PKM | 531 | 57,980 | 1.3270 | 0.4114 | 0.0091 |

0 ppm and 40 ppm represent in fermented goat milk with 0.00 mg L^-1^ and 40.00 mg L^-1^ benzoic acid concentrations, respectively.
